# Antibody Structure and Function: The Basis for Engineering Therapeutics

**DOI:** 10.3390/antib8040055

**Published:** 2019-12-03

**Authors:** Mark L. Chiu, Dennis R. Goulet, Alexey Teplyakov, Gary L. Gilliland

**Affiliations:** 1 Drug Product Development Science, Janssen Research & Development, LLC, Malvern, PA 19355, USA; 2 Department of Medicinal Chemistry, University of Washington, P.O. Box 357610, Seattle, WA 98195-7610, USA; dennisrgoulet@gmail.com; 3 Biologics Research, Janssen Research & Development, LLC, Spring House, PA 19477, USA; dr.alexey.teplyakov@gmail.com (A.T.); garygilliland911@gmail.com (G.L.G.)

**Keywords:** antibody engineering, therapeutic biologics

## Abstract

Antibodies and antibody-derived macromolecules have established themselves as the mainstay in protein-based therapeutic molecules (biologics). Our knowledge of the structure–function relationships of antibodies provides a platform for protein engineering that has been exploited to generate a wide range of biologics for a host of therapeutic indications. In this review, our basic understanding of the antibody structure is described along with how that knowledge has leveraged the engineering of antibody and antibody-related therapeutics having the appropriate antigen affinity, effector function, and biophysical properties. The platforms examined include the development of antibodies, antibody fragments, bispecific antibody, and antibody fusion products, whose efficacy and manufacturability can be improved via humanization, affinity modulation, and stability enhancement. We also review the design and selection of binding arms, and avidity modulation. Different strategies of preparing bispecific and multispecific molecules for an array of therapeutic applications are included.

## 1. Introduction

Currently, all antibodies and antibody-derived macromolecules being developed for a wide spectrum of therapeutic indications [1,2] require protein engineering. The engineering approaches being used are based on our knowledge of protein structure and, in particular, our knowledge of how the structures are linked to their function [3]. Our knowledge of the three-dimensional structure of antibodies has emerged from crystallographic studies reported from numerous laboratories beginning in the 1970s. At present, the Protein Data Bank (PDB) [4] contains over 3500 structures of antibody fragments (Fabs, Fvs, scFvs, and Fcs), as well as a small number of intact antibody structures. The structural data includes complexes of these molecules with proteins, other macromolecules, peptides, and haptens. The overall structure of antibodies, including the folding pattern of the individual domains and basic features of the antigen-combining sites, has been the subject of several reviews [3,5,6,7,8].

Human immunoglobulins are Y-shaped proteins composed of two identical light chains (LCs) and two identical heavy chains (HCs). In natural systems, the pairing of one LC with one HC associates with another identical heterodimer to form the intact immunoglobulin. The HC and LC of the heterodimer are linked through disulfide bonds. The two HCs of the heterotetramer are also linked by disulfide bridges. Human LCs can be one of two functionally similar classes, κ or λ. Both LC classes have two domains, a constant domain (CL) and a variable domain (VL). In comparison, human antibody HCs can be one of five isotypes, IgA, IgD, IgE, IgG, and IgM, each with an independent role in the adaptive immune system. IgAs, IgDs, and IgGs have three constant (C) and one variable (V) domains. IgEs and IgMs have one variable and four constant domains. The IgA and IgM isotopes have an additional J-chain, which allows the formation of dimers and pentamers, respectively. The other isotypes are monomeric (a monomer is defined here as a pair of HC-LCs.).

The general features of antibodies described below will focus on the IgG1 framework. Our knowledge of how antibody structure relates to function is being exploited to create antibodies and antibody-related biologics with the appropriate functional and biophysical properties to address specific therapeutic needs. The engineering approaches applied to antibodies, antibody fragments, antibody, and antibody fusion products include effector function engineering, antibody humanization, affinity modulation, and stability enhancement to improve efficacy and manufacturability.

### 1.1. Overall Features of the Immunoglobulin

**Figure 1:**
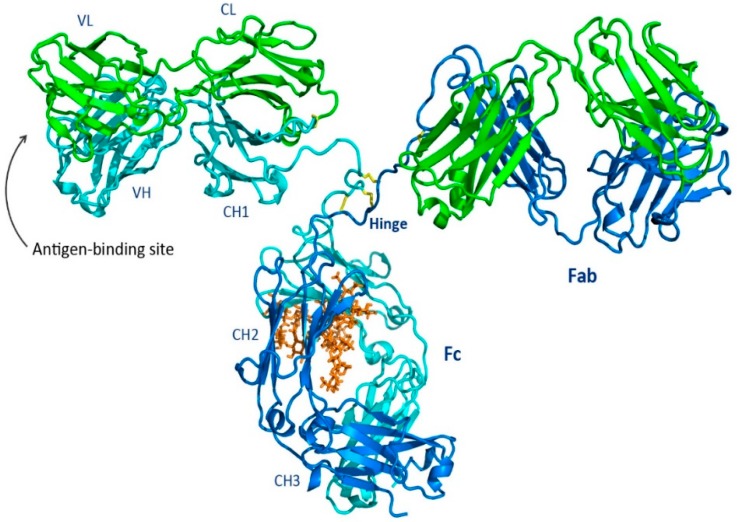
A ribbon representation of an intact IgG, Protein Data Bank (PDB) id: 1igt [11], which is a mouse IgG2a isotype. The light chains are green, the heavy chains are cyan and blue, the glycan is orange sticks, and the interchain disulfides are yellow sticks.

The intact antibody molecule shown in Figure 1 has three functional components, two Fragment antigen binding domains (Fabs) and the fragment crystallizable (Fc), with the two Fabs linked to the Fc by a hinge region that allows the Fabs a large degree of conformation flexibility relative to the Fc. Each of the Fabs have identical antigen-binding sites (or what is often called antigen-combining sites) for binding to a specific target antigen. The Fv region of the Fab is composed of a pair of variable domains (VH and VL) contributed by the HC and LC. In contrast, the glycosylated Fc region binds to a variety of receptor molecules providing the effector function profile that dictates how the antibody interacts with other components of the adaptive and humoral immune system.

**Figure 2:**
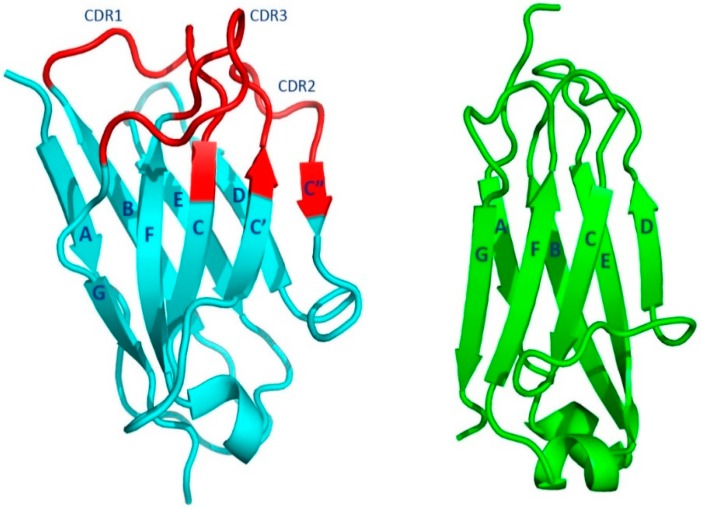
The immunoglobulin fold. The left ribbon image (cyan and red) of the heavy-chain variable (VH) domain illustrates the V domain immunoglobulin folding pattern (VH of Fab 388, PDBid 5i1a) [12]. The V domain complementarity-determining regions (CDRs) are shown in red. The right ribbon image (green) illustrates the similar folding pattern of a typical C domain (CL of Fab 5844, PDBid: 5i18 [12].

All the domains of heavy and light chains are approximately 110 amino acid residues in length whose conformations have been termed the “immunoglobulin fold” (Figure 2) [9,10]. The fold is comprised of two tightly packed anti-parallel β-sheets. One of the two β-sheets of the C domains has four β-strands, ↓A ↑B ↓E ↑D, and the other three β-strands, ↓C ↑F ↓G. The overall fold is often referred to as a Greek key barrel. The two β-sheets are covalently linked together by an intra-domain disulfide bridge formed between two cysteine residues in the ↑B and ↑F β-strands. The C domains are in general compact, with short loops connecting the β-strands. The two β-sheets pack together using the non-covalent interactions of the side chains of amino acid residues on the complementary faces.

The V domains of the immunoglobulin structure, which interact with the target antigen, are at the N-termini of the HCs and LCs. These domain structures are like that of the C domains but with some differences. The two β-sheets have a configuration like that found in the C domain. The four-stranded β-sheet, formed from four β-strands, ↓A ↑B ↓E ↑D is like the corresponding β-sheet in the C domain. The other β-sheet has five β-strands, ↓C’’ ↑C’ ↓C ↑F ↓B, instead of the three found in the C domain. An insertion of two β-strands, ↓C’’ ↑C’ is present between β-strands ↓C and ↑D. Just as in the C domain, an intra-domain disulfide bridge is formed between β-strands ↑B and ↑F. The less-compact V domains in general have longer loops connecting the β-strands.

### 1.2. Fab Region

#### 1.2.1. Fab Overall Features

The Fab regions of an immunoglobulin are formed by the pairing of VL and CL of the LCs with VH and CH1 of the HCs. The pairing of VL and VH, form the antigen-binding site. The two β-sheets formed with β-strands ↓C’’ ↑C’ ↓C ↑F ↓B pack together, forming a barrel-like structure that aligns the connecting loops (complementarity determining regions or CDRs, see below) and forming the antigen-binding site. In contrast, the CH1 and CL domains pack tightly in an almost perpendicular mode using the complementary faces of the opposite ↓A ↑B ↓E ↑D β-sheet.

The overall arrangement of the HC and LC domains of the Fab are characterized by what is called the elbow bend or elbow angle. This is defined by the angle between the pseudo-two-fold axes relating the two pairs of domains (VH, VL and CH1, CL) [10,13]. The switch region, an extended polypeptide chain, connects the V and C domains. The orientation of the V domains with respect to the C domains is referred to as the elbow angle or elbow bend, which can vary significantly. In an early survey of Fabs with kappa (κ) light chains, the angle was shown to vary from 116° to 226° [14]. Fabs with lambda (λ) light chains have a wider range of angles, indicating higher levels of flexibility. This may result from the presence of an extra amino acid residue (usually a glycine) present in the switch region of λ LCs. An early analysis of the elbow motion in Fabs discovered a conserved feature that is referred to as a molecular ball-and-socket joint [15]. This occurs in the HC at the interface between VH and CH1. The ball consists of conserved amino acid residues Phe148 and Pro149 in VH and the socket is formed by conserved amino acid residues Leu/Val11, Thr110, and Ser112 in the CH1 domain. This interaction could restrict the elbow angle to a maximum of 180°. However, larger angles were reported for subsequent Fab structures (e.g., [16]) in which the ball and socket move apart, allowing elbow angles >180° [14].

#### 1.2.2. The Fab Antigen-Binding Site

**Figure 3:**
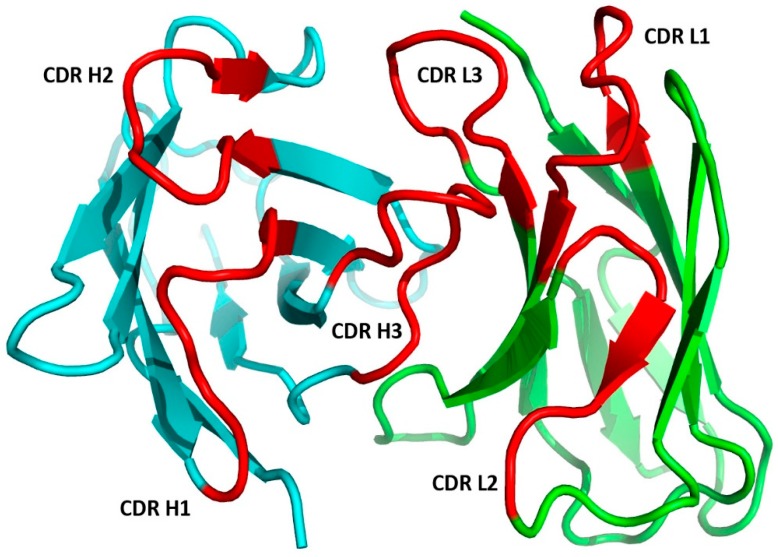
The Ab Fv region with the VH in cyan and the VL in green. The Martin CDRs are highlighted in red (Fv of Fab 388, PDBid: 5i1a) [12].

The antigen-binding site is formed by the pairing of the Fab VH and VL with the N-terminal region designated as the Fv region. As shown in Figure 3, each domain contributes three complementarity-determining regions CDR-L1, CDR-L2, and CDR-L3 for VL and CDR-H1, CDR-H2, and CDR-H3 for VH. These hypervariable regions were identified by early amino acid sequence variability analyses [17,18] that pre-dated our knowledge of the structure of the antibodies. The six CDR loops are in proximity to each other, resulting from the orientation of VL and VH after the formation of the Fv. This is a result of the packing of the β-sheets composed of the ↓C’’ ↑C’ ↓C ↑F ↓B from the two domains. This configuration brings the three CDRs of the VL and VH domains together to form the antigen-binding site. The strands of the two β-sheets and the non-hypervariable loops are referred as to framework regions (FRs).

Both the number of amino acid residues and the sequences can vary for the CDRs. Genetic recombination of the V, D, and J gene segments for VH and V and J gene segments for VL with subsequent somatic hypermutation in mature B cells accounts for antibody CDR sequence diversity. In the two domains, the CDRs are composed of amino acid residues in the loops connecting the framework β-strands ↑B and ↓C for CDR-L1 and CDR-H1, ↑C’ and ↓C’’ for CDR-L2 and CDR-H2, and ↑F and ↓G for CDR-L3 and CDR-H3.

The Fv amino acid residues in contact with the antigen have been called specificity-determining residues (SDRs) [19]. Antibodies in complex with haptens, proteins, or peptides show distinctive SDR patterns [19,20]. Anti-hapten antibodies have small and deep binding pockets at the VH–VL interface. The antigen-binding sites specific for peptides are groove-shaped depressions between VH and VL, while anti-protein antibodies tend to have extended and larger binding sites compared to those of the other two classes of antibodies. These structural features of antibody recognition sites for different classes of antigens have been employed in the development of productive synthetic antibody libraries for the specific recognition of haptens [21], peptides [22], and proteins [23].

#### 1.2.3. Relationship between Binding and Affinity

The antigen binding of antibodies often results in conformational changes in the contact surface areas of both the antibody and the antigen. These events have been studied in detail by many laboratories in the structure determinations of both an antibody fragment (Fabs or Fvs) alone and in complex with its antigen (for reviews, see [8,24]. When discussing antigen–antibody interactions, the general modes of binding are cited: Lock and key, induced fit, and conformational selection. In the lock and key model, the two molecules interact in a manner that minimizes changes in the conformations of the two protein surfaces from that observed in the unbound and bound states. Thus, the backbone conformations of the antibody and antigen are essentially the same in both the unbound and bound states. In contrast, the conformational changes for the antibody and antigen in the induced-fit mode can be quite extensive. Both the side chain and backbone atoms in the contact region can undergo conformational changes after the binding takes place, especially in the CDR regions. Of all the CDRs, the CDR-H3 most often has changes in conformations when the unbound and bound structures are compared. In addition, differences in the orientation of VL with respect to VH are often seen. Lastly, the Fab elbow angle may differ in the two forms. It has been suggested that the induced-fit mode of binding introduces plasticity into the antigen-binding site, expanding antibody diversity beyond that resulting from amino acid residue changes [25]. In the conformational selection model, the antigen samples a population of different conformational states prior to binding [26,27]. Antibody binding can then depend on pre-activation states of the antigen, which can be affected by the microenvironment around the antigen [28]. Sorting out the kinetics of target engagement also provides a guideline of how to optimize pharmacology. Understanding this aspect of binding can drive the development of better *in situ* antibody therapeutic design [29]. This also serves as a reminder that binding affinity may not be directly linked with pharmacology [30].

#### 1.2.4. Canonical Structures of the CDRs

An early structural analysis of antigen-binding sites of the small set of structures of immunoglobulin fragments available at the time revealed that the conformations of five out of the six hypervariable loops or CDRs had a limited set of main-chain conformations or ‘canonical structures’ [31,32]. The canonical structure model implied a paradigm shift in the field, replacing the notion that each antibody has unique hypervariable loop conformations. A canonical structure is defined by the loop length, the conformation of the loop, and the conserved amino acid residues within the hypervariable loop and FRs. Based on this model, studies of antibody sequences indicated that from the total number of possible combinations of canonical structures only a few occur [33,34,35]. This suggested that structural restrictions at the antigen-binding site may affect antigen recognition. Subsequent work [36] reported that the hypervariable loop lengths are the primary determining factor of the antigen-binding site topography, as they are the primary factor determining the canonical structures [31,37].

This early work was extended to include conformational analysis of the CDRs of 17 high-resolution antibody fragments [37]. The CDRs of the light chain CDR-L1, CDR-L2, and CDR-L3 were all found to have preferred sets of canonical structures based on the length and amino acid sequence composition. This was also found for CDRs of the heavy chain CDR-H1 and CDR-H2, but not for heavy chain CDR-H3, which is the most variable in length and amino acid sequence. This limited set of CDR canonical structures was included in macromolecular modeling strategies for antibody structures [31,32]. The early assignments of canonical structures have been extended using an algorithm that clusters the CDRs from a set of antibody fragments with low temperature factors and low conformational energies [38]. The results are frequently updated and available online (http://dunbrack2.fccc.edu/PyIgClassify/default.aspx) from the Dunbrack Laboratory.

#### 1.2.5. CDR-H3

One of the CDRs, CDR-H3, has a large range of lengths and amino acid sequence diversity and usually plays a primary role in the antibody–antigen interactions. The CDR-H3 conformation is quite variable in nature and canonical structures were not defined in the early cataloging efforts. In later studies, the residues in the loop nearest the framework (torso) and residues in the extended region of the loop (head) have been found to have defined conformations [39,40,41]. One interesting discovery by this work was that the backbone of the CDR-H3 base region can have either an ‘extended’ or ‘kinked’ conformation. The kinked conformation is a beta-bulge in the backbone of the stem region. In early studies of CDR-H3 structures, the kinked form was more prevalent than the extended one [41]. A recent study reported 16 representative Fab structures of a germline library, all having the same CDR-H3 amino acid sequence [12]. In fourteen of these structures, CDR-H3s were found in the kinked conformation, whereas in two structures CDR-H3s were in the extended conformation. This finding supports the hypothesis that the CDR-H3 conformation is controlled both by its sequence and its environment [42].

#### 1.2.6. Antibody Modeling

The knowledge of canonical structures enabled the development of antibody modeling (Fv region) [43]. In therapeutic antibody development programs, where the number of candidates being considered far exceeds the capacity of the crystallographic structure determination process, antibody modeling has become increasingly more important. Because of this need, approaches for antibody modeling continue to evolve along with the field of protein structure prediction. Recently, antibody modeling assessment studies have been undertaken to gain insight into the quality of the results of antibody structure prediction software. These blinded studies [44,45] involved providing the antibody structure prediction software groups with the sequence of Fv regions for which structures had been determined but were not yet publicly available. Once the predictions were completed by the participants, the results were submitted to the organizers and the models were assessed and compared with the unpublished structures. In the second study [45], after the prediction of the structures of the entire Fv were completed, the participants were provided with the Fv structures without their CDR-H3s. The structures of the CDR-H3s were then predicted and submitted. This was done to assess whether more accurate structures of CDR-H3 could be predicted if the context (the Fv structural environment) was provided. The participants included Accelrys, Inc. [46], Chemical Computer Group (CCG) [47], Schrödinger [48], Jeff Gray’s lab at John Hopkins University [49] Macromoltek [50], Astellas Pharma/Osaka University [51], and Prediction of ImmunoGlobulin Structure (PIGS) [52,53]. While only Accerlys, Inc. and Chemical Computer Group (CCG), and PIGS participated in the first assessment, all other aforementioned parties participated in the second assessment. In both studies, all the antibody modeling methods produced similar and reliable models for the FR, but with some exceptions in the CDRs. Each of the methods applied in these studies had different strengths and weaknesses. Overall, the second antibody assessment revealed an improved quality of the models with an incremental improvement in the accuracy of the predictions from the first assessment, but further development to improve these methods is clearly warranted [54].

### 1.3. Fc Region

In the 1950s, it was discovered that proteolysis of intact IgGs with papain produced large fragments about a third of the size of the intact molecule [55,56], and it was eventually discovered that one of the fragments could bind antigen and act as an inhibitor to the binding of the intact antibody. This turned out to be what we call today the Fab fragment. Another fragment approximately the same size turned out not to inhibit binding, and it was easily crystallized [57]. This crystallizable fragment is what we call the Fc. The structural features of this region of the antibody were defined in the initial structure determination of the human IgG1 Fc [58] and they have remained constant as the structures of many other Fcs have been determined (see a partial list of Fc structures in Teplyakov et al., 2013 [59]).

**Figure 4:**
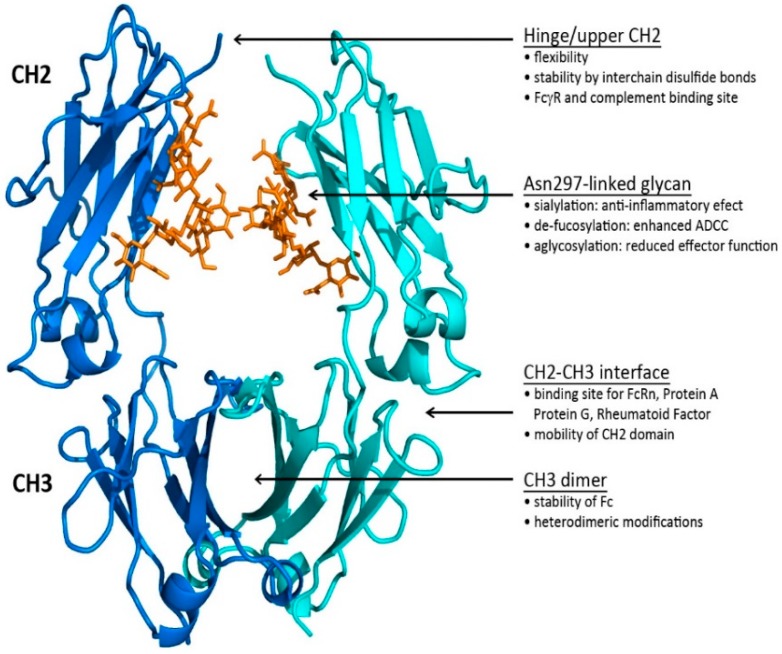
The structural features of the human IgG1 Fc and how they impact functionality. The Fc is represented by a ribbon image of the Fc structure (PDBid: 3ave [65]). The two heavy chains are shown in blue and cyan; the carbohydrate is represented by orange sticks.

The three-dimensional structure of the Fc [58] revealed how the two constant domains, CH2 and CH3, of the each of the HCs interact with one another (see Figure 4). The CH3s pack tightly with each other while the CH2s have no observable protein–protein contacts with one another. Rather, the space separating the CH2s is filled in part by the carbohydrate attached at Asn297. In some structures, the two carbohydrate chains interact through hydrogen bonds, either directly or through bridging water molecules. The flexibility imparted to the CH2s contributes to their role in the interaction with C1q and the FcγRs. The Fc region of an IgG can engage with Fc gamma receptors (FcγR) and the first subcomponent of the C1 complex (C1q) to mediate antibody-dependent cellular cytotoxicity (ADCC), complement-dependent cytotoxicity (CDC), antibody-dependent cellular phagocytosis (ADCP), trogocytosis, induction of secretion of mediators, and endocytosis of opsonized particles, as well as modulation of tissue and serum half-life through interaction with the FcRn [60,61,62]. The Fc has been the focus of significant engineering to modulate effector function activities found on monocytes, macrophages, dendritic cells, neutrophils, T and B lymphocytes, and natural killer cells [63]. Since there is often an interchange of mAbs coming from different mammalian forms, a systematic comparison of human Fc binding to mouse, cynomolgus, and human FcγRs have been made to correlate in vitro and in vivo Fc activity [64].

#### 1.3.1. The Fc CH2–CH3 Interface

The Fc CH2–CH3 interface has been recently characterized in a report of the structures of two crystal forms of the IgG2 Fc [59]. The interface is dominated by non-covalent interactions between the two domains supplemented by the presence of ordered water molecules. When the structures were compared with the structures of homologous IgG1 Fcs [65,66,67], it was observed that the CH2s change position relative to the CH3s. Further analysis revealed an Fc ball-and-socket joint between CH2 and CH3 that allows the CH2 domain to pivot around its Leu251 side chain, which is buried in a pocket formed by CH3 residues Met428, His429, Glu430, and His435. The movement of the CH2s is constrained by residues from both domains found at the CH2–CH3 interface and the hinge region. This Fc ball-and-socket joint is analogous to the one that is found in the Fab structures mentioned above [14,15,68], but in the Fc case, it is reversed relative to that found in Fab regions with the ball in the CH2 domain and the socket in CH3. The subset of CH2–CH3 interface residues associated with the Fc ball-and-socket are highly conserved among human IgG1, IgG2, IgG3, and IgG4 [59], indicating that it is a general structural feature that facilitates the motion of CH2 relative to CH3 in human IgGs. The positions of the amino acid residues at the interface vary as the domains change their relative orientation to one another, increasing or decreasing the gap between the domains. As the domains move, the water structure associated with the CH2–CH3 interface also adjusts. Future Fc engineering efforts can consider altering residues associated with the Fc ball and socket that could impact the flexibility of the Fc, potentially altering effector function activity.

#### 1.3.2. The Fc CH2 Carbohydrate

**Figure 5:**
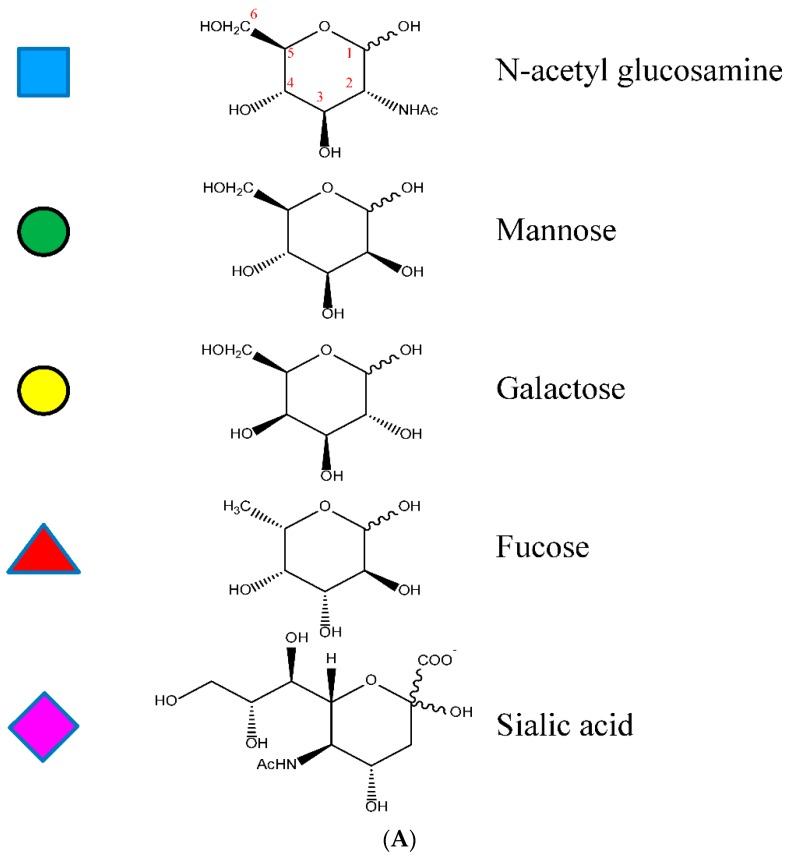

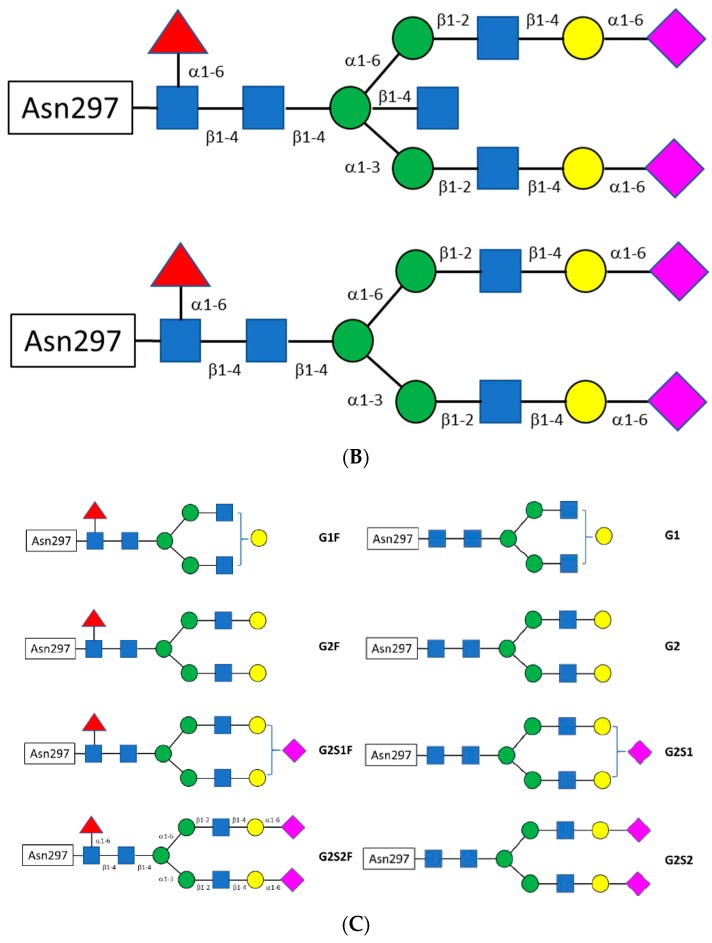
(**A**) Schematic representation of the most abundant recombinant N-linked oligosaccharide from human IgG Asn 297 (G2S2F) with glycosidic linkages. A similar representation of recombinant human IgG1 G2S2F is shown. The monomeric saccharides are shown as blue squares as N acetyl glucosamine; green circles as mannose; yellow circles as galactose; red squares as fucose; and purple rhombi as sialic acid or N acetyl neuraminic acid. (**B**) The glycosidic linkage numbers for representative oligosaccharides. The numbering of the glycosidic linkages are shown for oligosaccharides found in IgG molecules. The 1-4 N acetyl glucosamine can be found in human IgG structures. (**C**) Major species of N-linked oligosaccharides found in recombinant IgGs expressed in Chinese hamster ovary (CHO) cells may vary considerably by the addition of other sugar residues, such as sialic acids, N-acetylglucosamines, and galactose.

The Fc CH2 carbohydrate covers a hydrophobic face of the domain and helps to fill the void between the two HC CH2s. Each of the domains has covalently bound carbohydrate with the structure described in Figure 5. This structure may vary considerably by the addition of other sugar residues, such as sialic acids, N-acetylglucosamines, and galactoses, and in some cases, the absence of fucose [69]. The presence of the glycans contributes to the biophysical stability of the protein structure [70]. Several Fc crystal structures with different glycoform variants [65,71,72] and aglycosylated forms [73,74] have been reported. In these structures, the composition of the carbohydrate dictates the separation distance between the CH2s. The composition of the carbohydrate of the Fc can substantially influence the effector functionality of the antibody as well as the pharmacokinetic profile [75].

### 1.4. Hinge

The HC polypeptide region bridging CH1 and CH2 is called the hinge region and functionally allows the Fabs a large degree of conformational flexibility relative to the Fc. This facilitates the Fabs binding to multiple targets and allows the Fc to interact independently with other components of the immune system [76]. Structural knowledge of the IgG hinges is based upon the structures of intact mAbs, of Fcs, and of Fc:FcγR complexes. A review of structures deposited in the PDB [4] now reveals that there are 7 intact antibody structures, 87 Fc structures, and 15 FcγR complexes. There are ongoing efforts to utilize individual particle electron tomography to determine the diversity of conformational changes [77].

**Figure 6:**
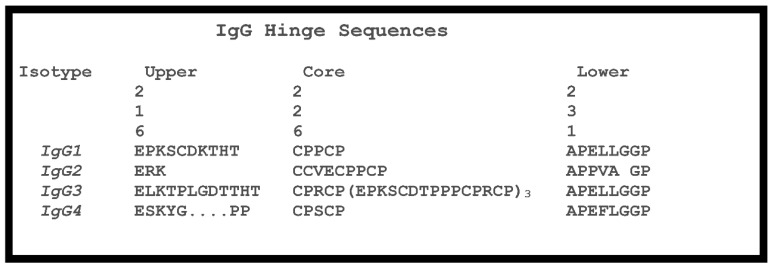
The hinge sequences of human IgG isotopes illustrating the upper, core, and lower hinge regions. Sequence numbers are given for the IgG1 hinge.

The antibody hinge can be divided into three regions, the upper hinge, core hinge, and lower hinge, each with a different functional role [66] (see Figure 6). On the N-terminal side, the upper hinge allows the movement and rotation of the Fabs. The central core hinge contains a variable number of cysteine residues depending on the IgG subtype that forms disulfide bonds, stabilizing the association of the HCs. On the C-terminal side is the lower hinge that allows movement of the Fc relative to the Fabs and whose amino acid residues can be involved in FcγR binding.

The hinges of Human IgG subtypes vary significantly in the number of residues and the number of possible disulfide bridges between the two heavy chains. This contributes to the overall stability of the antibody. For example, of all the IgGs, IgG4 is the only subtype that undergoes natural Fab-arm exchange producing antibody molecules that are bispecific [78]. In addition, this variability, including the differences in amino acid sequence, contributes in part to the strength of the interactions of IgGs with FcγRs.

An aspect of stability for antibodies and the hinge region is protease sensitivity. Papain [57] and other proteases [79] are used to cleave the upper hinge of IgGs, generating Fab and Fc fragments. Cleavage of the lower hinge single leads to single-clipped IgG or a double-clipped IgG with F(ab’)_2_ and Fc fragments. In humans, this cleavage can take place during inflammation, in tumor micro-environments or during bacterial infection by matrix metalloproteases, such as MMP-3, MMP-12, and MMP-7(matrix metalloproteases), and others like cathepsin G, GluV8, pepsin, and IdeS [80]. Mutations in the IgG1 [81], IgG2 [82], and IgA [83,84] hinge regions can mediate some levels of resistance to such enzymes. Such mutations can prevent hinge clipping to preserve the Fc effector function of therapeutic Abs in the inflamed tissue environment.

## 2. Structure-Based Antibody Engineering

Nobody is perfect, and the same applies to antibodies. Molecular engineering aims to improve the biochemical and biophysical properties of the antibodies of interest to make them good therapeutics and convenient research tools. Methodologically, there are two strategies to achieve this goal. Rational methods are based on structural knowledge derived from X-ray crystallography, Nuclear Magnetic Resonance (NMR) spectroscopy, and in silico modeling, and typically lead to the generation of a small set of variants. In contrast to rational, empirical methods are based on generating large libraries by employing phage, ribosome, or yeast display and rely on screening to select the desired variants [85]. This section of the review is focused on rational methods to engineer the antigen-binding function of the Fab arm of the antibody. The enormous progress that has been achieved in modifying the Fc-related effector function of the antibody has been reviewed recently [86,87,88] and will be discussed later in this review.

The availability of the three-dimensional structure of the antibody–antigen complex or even Fab alone greatly facilitates the design of the antibody variants with improved characteristics. Advances of X-ray crystallography over the last two decades coupled with the modern molecular biology and protein purification techniques have transformed structure determination into a routine procedure that requires minimal time and effort. Continual adaptations of the downstream process have included alternative purification schemes [89]. The benefits of the structural knowledge are manifold. For humanization, it helps to identify the critical positions outside of the complementarity-determining regions (CDRs) that must be preserved and positions within CDRs that may be replaced. For affinity maturation, it may point to a residue, which is otherwise unlikely to be considered as a game changer. For solubility improvement, modifications of the hydrophobic patches on the antibody surface (often not apparent in the linear sequence) are required.

In addition to crystallography, NMR and recently cryogenic electron microscopy (cryo-EM) have evolved as complementary techniques to obtain 3-D structures especially of Fab–Ag and Ab–Ag complexes. In the absence of experimental structural information, homology models are often considered as a decent alternative. However, despite the obvious development in algorithms and computer power, the quality of antibody structure prediction, particularly regarding CDR-H3, remains inadequate, and the results of antibody–antigen docking are also disappointing [90]. While homology models cannot fully substitute the experimental data, they can initiate the process for in silico design and evaluation of antibody mutants. We review such applications below.

### 2.1. Humanization

Historically the first and perhaps the most frequent application of antibody engineering was to reduce the immunogenicity of therapeutic antibodies of murine origin [91]. A variety of non-human species, including rodents, chicken, and rabbits, are employed today escape tolerance to obtain antibodies against human targets. All such non-human antibodies require humanization. The simplest approach was to make a chimera by combining the variable domains of non-human antibodies with human constant domains to generate molecules with 70% human content [92]. In many cases, chimeric antibodies demonstrated reduced immunogenicity but still elicited some human anti-therapeutic antibody response [93]. To further minimize immunogenicity, a CDR-grafting approach was proposed by G. Winter and coauthors [94]. The procedure involves the transfer of CDRs from a non-human (very often murine) “parental” antibody to the scaffold of a human antibody. The method was initially applied to a murine anti-hapten antibody. The CDRs from the heavy-chain variable region of the mouse antibody were substituted for the corresponding CDRs of a human anti-myeloma antibody. Following this experiment, a similar procedure produced a humanized anti-lysozyme antibody D1.3 [95], proving that CDR grafting can be used for antibodies that recognize protein antigens.

Besides CDR grafting, alternative humanization methods based on different paradigms, such as resurfacing [96], super-humanization [97], or human string content optimization [98], have been developed. All of them require the analysis of the amino acid sequence to evaluate the potential impact of the amino acid substitutions on the antibody structure and function. Typically, a relatively small number of humanized variants are produced and tested for antigen binding and functional activity. If the variants fail to meet the functional criteria, a new cycle of design, modification, and characterization is carried out to improve binding.

First, we consider CDR grafting as the principle method of antibody humanization. The procedure involves three tasks: (1) Defining the boundaries of the CDRs for grafting, (2) selecting human sequences to be utilized as framework (FR) donors, and (3) identifying residues within human FRs that may need to be replaced to maintain antibody binding. Although the tasks may seem consecutive, they are interrelated, and in practice should be carried out together.

#### 2.1.1. CDR Definitions

Amino acid residues that constitute the CDRs were identified by Kabat [99] based on their high variability as compared to the other regions of the antibody (Figure 3). By analyzing the first crystal structures of Fabs, Chothia and Lesk [31] proposed a definition based on the conserved conformations of the antigen-binding loops named canonical structures. Accumulation of the structures of antibody–antigen complexes has led to the Martin CDR definition [100], which considers the involvement of residues in antigen binding. Symmetrical CDRs, where the N- and C-terminal residues are opposite each other in the structure, were used for the purpose of canonical structure classification [38] as implemented in the PyIgClassify database [101]. In comparison to the Martin definition, CDRs L2, H3, and H1 include an extra one, two, and three residues, respectively, at the N-terminal end of the CDRs. Universal schemes that are applicable to immunoglobulins, T-cell receptors, and major histocompatibility complex (MHC) molecules have also gained popularity [102,103,104]. A comparison of different CDR definitions is presented in Table 1.

For the purposes of CDR grafting, the choice of the CDR boundaries is free and not limited by the common definitions. However, two factors should be considered. First, the CDRs should be as short as possible to minimize the number of non-human residues. Second, the CDRs should include at least all residues in direct contact with the antigen. All definitions have advantages and disadvantages in terms of CDR grafting. The ImMunoGeneTics information system (IMGT) [104,105] rightfully includes residues 93 and 94 in CDR-H3 as they are very important for the CDR conformation. On the other hand, the IMGT convention excludes residues 35 and 50 from CDRs H1 and H2, respectively, although they are often involved in antigen binding. Considering all the pros and cons, the Martin definition is a good compromise (Table 1). Basically, it combines the Kabat and Chothia definitions and differs from them only in the heavy chain, where CDR-H1 includes all residues of Kabat and Chothia while CDR-H2 is seven residues shorter than that defined by Kabat. Those seven residues are in the loop between β-strands C” and D and are never directly involved in contact with the antigen. In the light chain, there are no deviations between Kabat, Chothia, and Martin CDR definitions.

Regardless of the choice of CDRs, about 20% of the residues that bind the antigen fall outside the CDRs [106,107]. Moreover, these residues are at least as important to antigen binding as residues within the CDRs, and in some cases, they are even more important energetically. Therefore, for CDR grafting, the CDR definition is a good starting point, but the framework residues interacting with the antigen must be considered. Typically, for shorter CDRs, more FR residues, and for longer CDRs, fewer FR residues will need to be considered for back mutations.

Residue numbering schemes evolved in parallel with the CDR identification and aimed at the correct positioning of insertions and deletions in the antigen-binding loops. Since the Chothia numbering scheme [32] was based on structural considerations, it represents the best choice and is widely used in many applications. An advantage of a universal numbering versus sequential is that all structurally identical positions are numbered identically, which is convenient for alignments and comparisons. The Chothia numbering of residues is used throughout this review.

#### 2.1.2. Human Germline Selection

The second step in the humanization process is to identify human FR donors. Initially, human antibodies of a known structure were used regardless of their homology to the non-human antibody in the so-called fixed FR approach [94,95,108]. Moreover, both VH and VL donors were often selected from a single antibody to ensure optimal pairing. However, this approach often resulted in a significant or even complete loss of affinity and was replaced with a method termed “best fit” [109], where human VH and VL sequences with the highest homology to the non-human antibody were selected. Comparison of the fixed FR and best fit strategies showed that the latter yields humanized antibodies with a higher affinity than variants obtained by the fixed FR method [110]. Another strategy of selecting human FRs as a template for humanization is by generating consensus sequences [111,112].

Regardless of the method chosen to select human FRs, there are two sources of human sequences: Mature and Germline. Mature sequences generated by the immune response carry somatic mutations [113] and therefore are potentially immunogenic. In contrast, human germline sequences are considered least immunogenic and have been recently used as FR donors almost exclusively [114].

The human repertoire consists of several dozen germline genes coding VH regions and approximately an equal number of VL genes, which are divided between κ and λ types [105]. Both heavy and light chain germlines are grouped into families according to sequence similarity. Among VH germlines, families 1, 3, and 4 are the most ubiquitous. The majority of λ VL germlines fall into families 1, 2, and 3, whereas the rest are distributed among families 4 to 10. Kappa VL germlines are almost entirely distributed over three families (1, 2, and 3) except for two genes, *IGKV4-1* and *IGKV5-2*, which represent families 4 and 5. The sequence identity within families is close to 90% while it can be as low as 50% for two germlines from different families.

Methods of human germline selection for CDR grafting are varied. One option is to base the choice on the overall sequence similarity between the non-human antibody and human germline within the variable domains. A more focused and more common approach considers sequence similarity only in the FR while neglecting the CDRs. The idea behind this is that homologous FRs provide the same scaffold for the CDRs and ensure their conformation, while the CDRs themselves are not changed at all (they are grafted). An alternative approach considers sequence similarity within the CDRs and relies on the canonical structures that are defined largely, although not exclusively, by the CDR sequence. The latter method is called super-humanization and will be discussed below.

Typically, a single germline is selected for the entire variable domain, one for VH and one for VL. However, one may apply a hybrid approach when a donor for each FR is selected independently, so that the resulting sequence will be assembled from different germlines [115]. This method has an obvious advantage of more flexibility and a potential for selecting human germlines with higher similarity score. It is believed, however, that mosaic constructs may exhibit impaired stability when compared to intact germline sequences owing to suboptimal VH-VL pairing and potential clashes in the core of the variable domain. Residues that come from different FRs may appear mutually incompatible when composed from different germlines.

A combination of sequence and structural criteria in the selection of human germlines was utilized in the humanization of mouse anti-glycoprotein VI Fab ACT017 developed for the treatment of arterial thrombosis [116]. The choice of templates for VH and VL was based upon the following four independent criteria: (1) Human germline sequences most similar to mouse germlines of the parental antibody; (2) high sequence identity and identical canonical structures of the CDRs; (3) high sequence identity and closely related CDR canonical structure; and (4) the same antibody template for both V-domains even at the cost of a less optimal template for one of the chains. Additionally, the human myeloma antibodies NEW (for VH) and REI (for VL) were selected because they are well-characterized in terms of stability and expression and they are frequently used in a fixed-FR strategy of humanization. Owing to some overlapping among the best candidates selected by applying these criteria, there were only 4 variants for each chain, resulting in a total of 16 VH-VL pairs. The 16 Fabs were expressed and evaluated for antigen binding, and only four of them showed the desired level of binding. One of the binders was based on bevacizumab, the FR donor for both VH and VL according to the selection criteria (4). The requirement of the identical canonical structure worked well only for VH and produced the best variant, which had the light chain FR from human antibody REI. Curiously, none of the variants using the VH template with the human germline most like the mouse parent retained significant binding activity.

#### 2.1.3. VH–VL Pairing

When selecting suitable human germlines for the heavy and light chains, considerations of the VH–VL pairing is important from two points of view. Firstly, selected germlines should form a stable Fv, and secondly, the mutual orientation of the VH and VL domains should correspond to that observed in the parental antibody. While the first requirement seems obvious, the second is debatable. A completely opposite reasoning, namely that the VH–VL interface should preserve the interactions of the donor FRs, was indeed utilized (without success) in some studies [117].

The importance of maintaining the VH–VL orientation during the humanization process was demonstrated in the study of anti-lysozyme murine antibody HyHEL-10 [118]. Following humanization, the affinity dropped 10-fold. Structural analysis indicated that all interactions between antibody and antigen were conserved; however, the relative orientation of VH and VL had changed. Amino acid differences between the mouse and humanized mAbs were then mapped onto the structure. In two positions in the FR of the heavy chain, there were rather unusual residues K39 and Y47 in the parental antibody that were replaced during humanization by the conserved Q and W, respectively. A single back mutation W47Y in the humanized mAb completely recovered the affinity. The double mutant W47Y/Q39K showed a further two-fold improved affinity. The crystal structure of the final variant confirmed the VH–VL orientation to be exactly as in the parental antibody.

Early studies have established the promiscuous nature of VH and VL pairing [119,120,121]. The remarkable ability of the human antibody repertoire to adapt to a specific target by generating a highly diverse panel of antibodies was recently demonstrated by analyzing antibodies raised against a single protein, B-lymphocyte stimulator [122]. Over 1000 antibodies, all different in amino acid sequence, have utilized 42 functional VH, 19 λ, and 13 κ VL germlines. Analysis of the sequences revealed that a given VH sequence can pair with many light chain sequences of both λ and κ types.

Another and much broader study included over 800 different antibodies generated against 28 clinically relevant antigens and isolated from human B cells from 160 donors [123]. Nearly all possible functional germlines (45 VH, 28 λ VL, and 30 κ VL) were represented in the experimental set. The V gene usage indicated no strong bias toward any VH–VL pairing. However, the VH1-λ VL1 germline family pairings were preferentially enriched and represented a remarkable 25% of the antigen-specific selected repertoire.

Somewhat contradictory to previous observations was the conclusion from the analysis of a large dataset of paired light and heavy chains from the Kabat database (Kabat et al., 1991) that VH–VL pairing does not occur at random [124]. Apparently, germline pairing preferences do occur in human antibodies, but only for a small proportion of germlines. The VH1 family shows a strong preference for VK3. On the other hand, no correlation was found between the germline pairing and the VH–VL packing angle.

Although the total number of human germlines and hence the VH–VL pairs is quite limited, relative affinities for each possible pair have not been tabulated. A major reason is that CDR-H3 forms a significant part of the VH–VL interface and therefore affects the pairing potential. In other words, the pairing propensity of any two given germlines depends to a large extent on the sequence and conformation of CDR-H3, which is highly variable. However, for a given CDR-H3 (as is the case in humanization), the differences between various pairings may be substantial.

An interesting, albeit limited, study of VH–VL pairing has reported thermostability values of a panel of 16 Fabs that were produced by all combinations of four VH germlines and four VL (κ) germlines with a fixed CDR-H3 [12]. It was found that the melting temperatures (T_m_) of the Fabs differed by more than 20 degrees. For each given light chain, the Fabs with germlines IGHV1-69 and IGHV3-23 are substantially more stable than those with germlines IGHV3-53 and IGHV5-51. Germline IGKV1-39 provides a much higher degree of stabilization than the other three light chain germlines when combined with any of the heavy chains. These results indicate that the selection of the right VH–VL pair is of prime importance during humanization.

**Figure 7:**
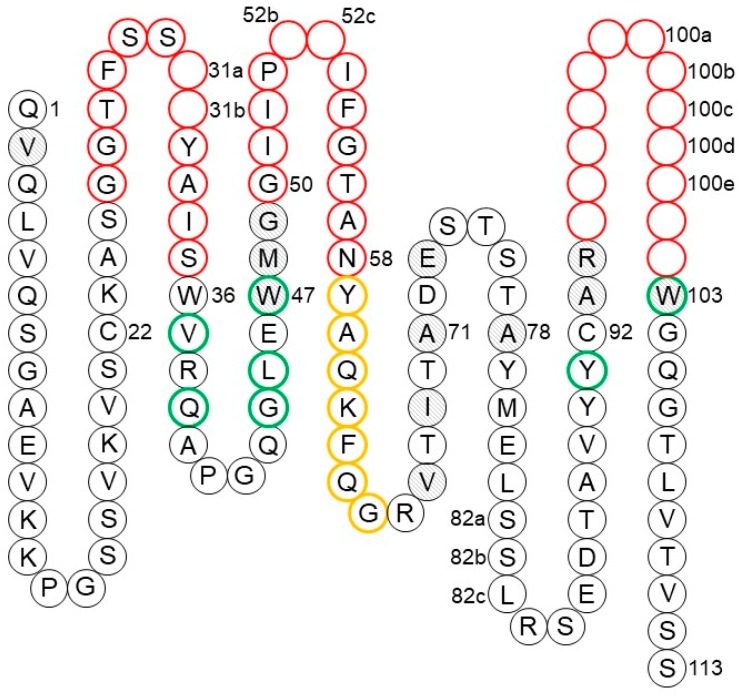
Collier de Perles presentation [105] of VH showing CDRs (red), Vernier zone residues (gray), and VH–VL interface residues (green). Amino acids correspond to human germline IGHV1-69*01 with the Chothia numbering of residues.

**Figure 8:**
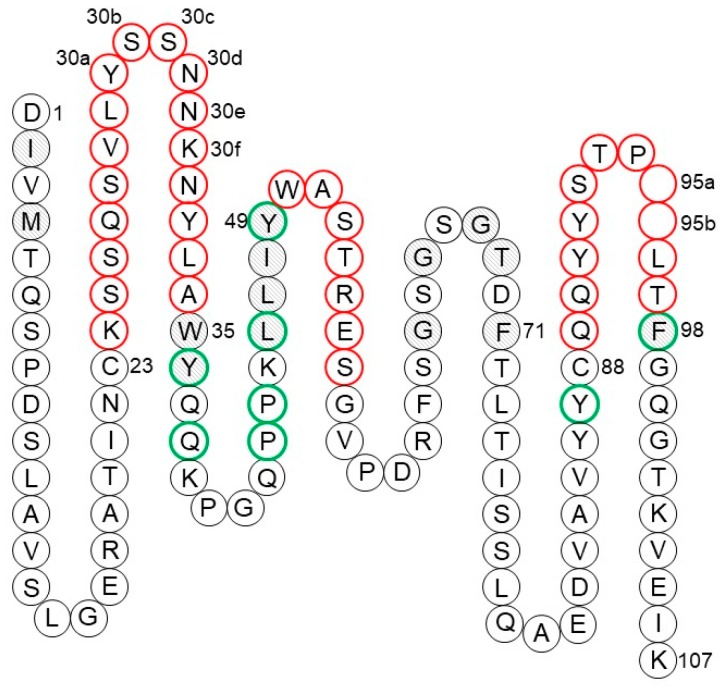
Collier de Perles presentation [105] of VL showing CDRs (red), Vernier zone residues (gray), and VH–VL interface residues (green). Amino acids correspond to human germline IGKV4-1*01 with the Chothia numbering of residues.

For a given CDR-H3, the task of selecting the optimal VH–VL germline pair is reduced to the preservation of residues at the VH–VL interface. This is an additional consideration for human germline selection besides the sequence similarity to the parental antibody. Since the CDRs are grafted as they are, only a small set of FR positions should be considered. These positions may be deduced from a simple analysis of the VH–VL interface in crystal structures. The minimal set of VH–VL interface residues includes seven residues from VH and eight residues from VL (Figure 7 and Figure 8). Besides two residues flanking CDR3, all other residues are in FR2 in both VH and VL. Most of these residues are conserved between human and mouse germlines and across human germlines. However, in those cases when they are different, either because of human vs. mouse differences or due to somatic mutations, the so-called back mutation may be required, as discussed below.

Computer programs predicting the VH–VL packing may provide some guidance in finding the best pair of germlines. Several tools have been developed recently along with the realization of the VH–VL orientation as a key parameter in antibody humanization and antibody modeling in general. A straightforward but effective approach has been implemented by Narayanan et al. [125], who used side-chain rotamer sampling for the interface residues followed by molecular mechanics energy calculations. The original main-chain conformations were from the crystal structures. A similar approach was implemented in the Rosetta Antibody modeling software [126].

A machine-learning approach to predict the VH–VL packing angle has been developed and trained on sets of interface residues taken from 567 crystal structures [127]. Rather than selecting interface residues for predicting the packing angle, a genetic algorithm was used to perform feature selection. It was designed to select a maximum of 20 interface positions that were optimal in training the neural network. Thirteen positions were identified as the most influential in determining the packing angle. The results showed an approximately normal distribution of errors with a half width at half maximum of about 2°, which is within the error observed in the crystal structures of antibodies [54].

Yet another approach for determining the VH–VL orientation [128] is also based on the identification of important residues. To describe the VH–VL orientation, six measures (five angles and a distance) were used. Correspondingly, six sets of key positions were identified, with few overlaps between them, 35 positions in total (24 in VL and 11 in VH). To consider so many positions in germline selection is impractical. Instead, the VH–VL packing orientation in the humanized variant may be predicted with one of the computational tools and compared to that of the parental antibody.

#### 2.1.4. Back Mutations

Straightforward CDR grafting may result in reduced target binding even if the VH–VL interface residues are preserved. This problem often arises when non-human CDRs and human FRs are mutually incompatible. Therefore, any CDR grafting protocol must include a step to identify FR positions that are critical for maintaining the CDR conformation. In germline selection, these critical positions should have higher priority than the overall sequence similarity because of their direct impact on the CDR conformation. If a non-human residue in a critical position cannot be preserved because there are no such human sequences, one usually applies the so-called back mutation, i.e., a mutation of a residue in the human FR to the amino acid that occurs in the non-human parent. Such a mutation reduces the humanness score of the resulting variant, but the change should improve the binding affinity.

Foote and Winter [129] identified 30 residues underlying and in direct contact with the CDRs that potentially influence CDR conformations. These residues constitute the so-called Vernier zone. Four of them, heavy chain residues 27–30, are considered part of CDR-H1 in the Chothia, Martin, and IMGT definitions (Table 1). The remaining 26 residues are divided equally between VH and VL (Figure 7 and Figure 8). One of the most recognized examples of a Vernier zone residue is position 71 of the heavy chain that defines the canonical structure of CDR-H2 [130]. Humanization of a few mouse antibodies, including anti-lysozyme mAb D1.3 [95], anti-acetylcholine receptor mAb 198 [131], and anti-tumor-associated glycoprotein mAb B72.3 [132], illustrates the importance of preserving the original residue in this position. However, this is not always the case. For instance, residue 71 was not a major factor in the humanization of anti-cytomegalovirus mAb 37 [133]. Similarly, substitution of Arg for Ala71 during humanization of anti-tissue factor mAb 10H10 was also well tolerated [134]. Hence, one may conclude that the importance of each critical residue depends on the involvement of different CDRs in antigen binding.

Unfortunately, it became a common practice to back mutate most of Vernier zone residues, just to reduce the possibility of a negative impact of human residues on binding [135,136]. However, this will inevitably add several ‘non-human’ residues to the humanized antibody. Together with CDRs, this may amount to 40% of residues in the variable domains of the antibody, which can hardly be called human. Therefore, a careful analysis of the importance of each Vernier zone position in the context of given CDRs and antibody–antigen interactions is the cornerstone of the humanization process. The availability of the crystal structure of the antibody–antigen complex greatly facilitates the design of humanized variants as it instructs on the FR positions that are indeed critical for antigen binding.

Computer modeling may to a certain extent replace the experimental structure, particularly in the regions at the periphery of the binding site where germline sequences and canonical structures dominate the landscape. The central zone around CDR-H3 remains problematic for accurate modeling, which was confirmed by the latest antibody modeling exercise [54]. Besides the limitations of antibody models, the lack of information on the CDR involvement in antigen binding often leads to an excessive number of back mutations in the humanized antibodies. To avoid such outcomes, each potentially critical position should be tested for back mutation and only those mutations that affect binding should be incorporated into the final antibody.

Back mutations may be applied not only to restore the binding affinity but also to improve the expression of the humanized variants. In the course of humanization of anti-lysozyme scFv F8, it was noticed that FR substitution of Y90F in the VH domain dramatically reduced the bacterial expression of all variants [137]. The back mutation in this position restored the expression and yielded a stable and fully functional antibody. Alternatively, there have been efforts to minimize the affinity of certain Fab domains by introducing more germline sequences [138]. This has been used to increase the potential toxicity of some binding arms.

#### 2.1.5. Deimmunization

While some positions in FRs may require back mutation, several positions within CDRs may be converted to human germline residues when they are not involved in the interactions with antigen or they do not influence the CDR conformation. There is no need to keep non-human residues in such neutral positions. This approach was used for the humanization of three mouse antibodies targeting CD25, vascular endothelial growth factor (VEGF), and tumor necrosis factor alpha (TNFα) [139]. Successive and iterative explorations of the human germline repertoire using semi-automated computational methods allowed the selection of functional humanized mAbs with the highest level of humanness. The resulting antibodies retain the potency of the corresponding chimeric mAbs and have in vitro activity comparable to that of their respective marketed drugs, daclizumab, bevacizumab, and infliximab.

The idea of incorporating human germline residues into the CDRs is related to the finding that CDRs are likely the only segments in humanized and fully human antibodies to contain CD4^+^ T-cell epitopes [140]. Analysis of a set of eight humanized antibodies representing different VH and VL regions from different genomic segments and affinity maturation processes indicated that prominent CD4^+^ T-cell epitopes are found only in CDRs and never in FRs. The immunogenic potential of the antibodies could be reduced while retaining their binding properties by incorporating just one or two amino acid substitutions within each T-cell epitope. The approach, which is termed deimmunization, may be considered as complementary to back mutations. It was successfully applied during humanization of an anti-prostate-specific membrane antigen mAb J591 [141], a therapeutic mAb specific for the protective antigen from *Bacillus anthracis* [142], and an antibody against the αv subunit of human integrin [143].

A structure-guided deimmunization method, called EpiSweep, was developed by Parker et al. [144]. The algorithm identifies sets of mutations in potentially immunogenic peptide fragments making optimal trade-offs between structure and immunogenicity, embodied by a molecular mechanics energy function and a T-cell epitope predictor, respectively. Although the program was developed for any therapeutic protein, apparently it may be used specifically for deimmunization of antibodies.

Regarding terminology, some authors consider chimeric antibodies with human constant domains as deimmunized antibodies [145]. We use this term here for a humanized antibody that was additionally modified to enhance the human content.

#### 2.1.6. Resurfacing

An alternative way of reducing immunogenicity risk of the humanized antibody is to replace only the surface residues in the non-human antibody with the residues present in human germlines [146]. Contrary to CDR grafting, resurfacing retains the non-exposed residues of the non-human antibody. This procedure is expected to eliminate potential B-cell epitopes while minimizing the perturbation of residues determining the antigen-binding properties of the antibody.

A systematic analysis of antibody structures was performed to determine the relative solvent accessibility distribution of residues in murine and human antibodies [96]. It appeared that residues in identical positions on the surface of human and murine variable domains are conserved with 98% fidelity across species. Thus, very few amino acid changes are needed to convert a murine Fv surface pattern to that of a human Fv surface.

The method was applied to two murine mAbs targeting CD56 and CD19 [147]. Two different procedures for selecting a human sequence were compared. For one mAb, a database of clonally derived human VL-VH sequence pairs was used while for the other, sequences for VL and VH were independently selected from the Kabat database [148]. Both resurfaced antibodies retained the affinities for their cell surface ligands.

Although most humanization projects in recent years have employed some version of the CDR-grafting method, resurfacing is still in use. For example, to reduce immunogenicity for clinical applications, mouse anti-CD34 mAb was humanized using the resurfacing approach [149]. The structural model was built using templates from the PDB to identify solvent-exposed positions for amino acid replacements with the threshold set at 30%. There were 28 solvent-accessible residues in VH and 35 in VL. Human germline sequences with the highest identity to mouse variable regions were identified, which led to amino acid substitutions in only four FR positions in VH and in five FR positions in VL. The resulting mAb retained the biological functions of the mouse mAb.

Similarly, a murine mAb, which specifically recognizes the pathogenic form of the prion protein, was resurfaced [150]. The design was based on sequence alignments and computer modeling and resulted in an scFv version bearing 13 mutations as compared to the murine parent. The deimmunized antibody demonstrated unaltered binding affinity and specificity. This is not surprising since resurfacing introduces a minimal number of mutations that are located on the surface of the molecule and are unlikely to cause conformational changes in the variable domains. Therefore, retaining affinity is virtually guaranteed, which is not the case in the CDR-grafting humanization. However, the amino acid sequence of the variable domains remains essentially non-human and may present potential epitopes for MHC class II molecules regardless of their surface exposure. Presentation of the epitope peptides to T cells may cause their activation, leading to the induction of signaling pathways [151].

#### 2.1.7. Super-Humanization

Human FRs for CDR grafting may be selected in two different ways, by the highest sequence similarity in the FRs or within CDRs. In the second approach, the FR homology is irrelevant. This method was applied to the humanization of murine anti-CD28 antibody and was called super-humanization [97]. The donor FRs were selected from the human germline gene repertoire based on CDR canonical structures. The super-humanized antibody exhibited a 30-fold loss in affinity.

Another example involving the super-humanization of the murine anti-lysozyme mAb D1.3 was relatively successful. The affinity loss of super-humanized D1.3 was only six-fold [152]. In a final example, the application of the method to the murine mAb 1A4A1, which was raised against Venezuelan equine encephalitis virus, yielded an antibody that retained antigen-binding specificity and neutralizing activity [153]. However, given the mediocre results of the method, it has not gained popularity.

It should be noted that the term super-humanization has also been used in a different sense, particularly when human or simian antibodies contained somatic mutations in FRs and were modified to increase their humanness, as measured by, e.g., the germinality index [154]. Obviously, no CDR grafting was needed in those cases, and super-humanization simply reflected a higher human content of the engineered antibody.

#### 2.1.8. Humanness Optimization

The humanness of the antibody can be assessed by any indicator that is able to distinguish human from non-human sequences. The human string content (HSC) score evaluates the proportion of human germline residues within a given sequence [98]. It can be calculated for a peptide in the target sequence by counting the number of residues identical to their counterparts in the most similar aligned peptide from a human germline. The validity of the HSC score was confirmed by analyzing 513 murine, 32 chimeric, 61 humanized, and 279 human antibody sequences from the IMGT database [155]. Human and humanized sequences produced significantly higher HSC scores when compared to murine and chimeric antibodies. Interestingly, the light chain scores were higher, perhaps due to relatively less diversity among light chains than among heavy chains. The HSC may be used for antibody humanization by maximizing the score rather than using a global identity measure to generate multiple diverse humanized variants. The method was successfully applied to the humanization of four antibodies with different antigen specificities [98]. The resulting variable domains differ fundamentally from those of CDR-grafted antibodies since they are immunologically more human because of being derived from several discrete germline sequences.

Because HSC optimization derives local information from multiple germlines, consideration of three-dimensional information is prudent to avoid clashes. A computational filter that screens for mutual compatibility of different fragments, called analogous contact environments (ACE), evaluates structural patches of amino acids for precedence in a database of antibody sequences [155]. For a given position, the structural precedence score measures the degree of match, weighted for distance and similarity, to the most homologous patch in the database. Averaging over all residues provides the global structural precedence for the sequence. Although a low precedence value does not necessarily mean low structural viability, a higher precedence value indicates that similar structural environments are sampled in the database, suggesting that the test sequence is more likely to behave favorably.

The humanness scores that are based on pairwise sequence identity between the sample and a set of germline human sequences may consider the average similarity [156], or the average among the top 20 sequences [157], or the highest similarity over windows of 9 residues [98,155]. In a different approach [158], the score function accounts both for local preferences and for pair correlations between residues at different positions. The method does not distinguish CDRs from FRs, which may be a plus since the latter may contain antigen-binding residues. Moreover, the relationship between the humanness score and the observed immunogenicity in patients was also considered [159].

With the growing wealth of sequence databases, statistical-inference methods could become an increasingly relevant tool, with a range of applications well beyond antibody humanization. Within a humanization protocol, the advantage of this approach over CDR grafting is that it proposes a set of candidate sequences, at increasing distance from the non-human parent toward the highest humanness score, instead of requiring the introduction of arbitrary back mutations.

### 2.2. Lambda to Kappa Chain Switching

Upon humanization, the type of the light chain of the parental antibody is usually not changed, i.e., if it was λ in the non-human antibody, the FR for a humanized variant is selected from the human λ repertoire. In some situations, switching the light-chain type may provide certain benefits. For instance, the production of bispecific antibodies from two mAbs might involve a purification step, which could be easily optimized if the mAbs contain light chains of different classes.

Technically, there are several significant differences between κ and λ chains that complicate the task. One is a deletion of a residue at position 10 in λ that is present in κ. Another is a different set of canonical structures for CDR L3, which are longer in λ chains and lack a conserved cis-proline at position 95. Also, λ CDR L1s differ from those of κ by being longer and fold into a helical structure. The overall sequence similarity between κ and λ germlines is below 50%. However, the FRs sequence similarity of around 60% of identical residues is higher than between some κ sequences (e.g., IGKV4-1 and IGKV5-2, which share only 51% identity in FRs). More importantly, all eight residues at the VH–VL interface are conserved between κ and λ types (Figure 8). Therefore, light-chain type switching seems feasible, and this has been confirmed in a few reports.

As part of a bispecific antibody, the Fab arm directed against FcγRIII was humanized by CDR grafting [117]. In a first attempt, the murine λ VL was converted to a humanized λ chain, which led to a complete loss of antigen binding and extremely poor folding efficiency. Initial humanization applied a fixed-FR approach using the human myeloma protein KOL as the FR donor, which had only 51% to 54% identity to the mouse antibody. Despite several back mutations in the Vernier zone, the strategy failed. Hence, the CDRs were transplanted onto a human κ light chain using the same strategy. Humanized anti-HER2 mAb 4D5, characterized by the VH subgroup III and VL subgroup I, was selected as an FR donor. Residues in positions 46, 49, 66, and 71 in VL and 71, 73, and 78 in VH were back mutated for various reasons. This resulted in a functional Fab, yet with a 100-fold decreased antigen affinity, which was subjected to affinity maturation through random mutations in both VH and VL. The optimized Fab exhibits an affinity within a factor of two from that of the original murine antibody. This required nine mutations, six of which are in VH and three in VL. Interestingly, most of the mutations occur at the VH–VL interface. Even if the humanization strategy was not optimal, it demonstrated that switching λ to κ may be successful.

Another attempt of the λ-to-κ switch occurred during optimization of an anti-GCN4 murine scFv [160]. The CDRs were grafted onto the FR of another murine scFv, which was selected due to its high stability. In this process, the CDRs of the parental λ VL were transferred to the FR of the donor κ VL. Homology modeling of the designed variant revealed some structural inconsistencies, particularly a potential clash between CDR L1 and loop 66–71 (sometimes referred to as the DE loop or CDR4). Therefore, this loop was back mutated to the original sequence. Additionally, eight residues in VH were also back mutated. The resulting scFv was significantly more stable than the original, but lost binding by three orders of magnitude. Back mutation of seven residues at the VH–VL interface to restore the proper orientation of the domains further enhanced the stability of the construct, which still had an order of magnitude reduction in the original scFv affinity.

A successful case of λ-to-κ conversion to improve the thermodynamic properties of scFv was reported recently [161]. The heavy chain of this scFv originated from the IGHV1-69*01 germline, whereas the light chain appeared to contain a fusion of two genes, IGLV3-19*01 and IGLV1-44*01, likely resulting from PCR aberration during library construction. The idea to replace the λ light chain by κ IGKV3-20 was based on the observation that this germline commonly pairs with IGHV1-69 to give highly expressed stable antibodies [162]. To guide the design process, a homology model of the converted scFv was constructed that revealed a potential clash between CDR L1 and loop 66–71. Analysis of a large set of PDB structures confirmed that this problem is typical for a λ-to-κ conversion. To facilitate CDR grafting, the DE loop from the original antibody was retained. No back mutations were necessary in VH. The resulting scFv showed increased thermostability and expression levels while retaining the binding affinity to the target. The scFv variant with the κ DE loop was less stable while also retaining binding.

These results indicate that λ and κ chains may be swapped without compromising the functional properties of the antibody. This strategy may be applied in antibody humanization or may prove useful for optimizing the biophysical properties of therapeutic candidates.

### 2.3. Affinity Maturation

Natural antibodies, both human and non-human, often do not possess the binding properties required for their therapeutic applications. This appears to be a consequence of the affinity ceiling that characterizes the mammalian immune system and B-cell responses [163,164]. Increasing the binding affinity is an important and almost inevitable step in the development of the lead candidate since it is related to the dose needed for treatment and the therapeutic efficacy. Different approaches, tools, and strategies are available and have been validated through the engineering of antibodies directed against various antigens. All of them can be divided into two groups according to the process of generating antibody variants. One is the rational design of the variants, followed by their expression in the system of choice. The other is the construction of a library of variants where several positions are diversified, followed by their display in a system of choice with the appropriate selection method. Owing to the large number of variants in a library covering the entire combinatorial space, the latter method is most commonly used for affinity maturation. In cases when only a few positions and a few amino acids are to be tested, perhaps the former approach may fulfill the task as it is fast and inexpensive. Whichever method is used, structure-based computational design may facilitate the process by in silico evaluation of the candidates to minimize either the library size or the number of mutants to be expressed.

A high-resolution structure of the antibody–antigen complex allows detailed analysis of the antibody–antigen interactions and greatly facilitates the design of affinity-enhanced variants. Even the structure of the Fab alone may instruct the selection of the most promising positions for mutagenesis. There are new developments of using NMR relaxation dispersion and hydroge-deuterium exchange experiments to map out regions for optimization of the affinity [165]. In the absence of any experimental information, computer modeling may fill the gap to a certain extent, and several successful examples based on theoretical models are discussed below.

First, we consider affinity maturation by structure-based rational design of the antibody variants with improved side chain packing and electrostatic interactions. A case in point is the improvement of the binding affinity of the anti-integrin antibody VLA1. Engineering increased the affinity by an order of magnitude primarily through a decrease in the dissociation rate [166]. Inspired by the crystal structure, a diverse set of single mutations (>80 variants) at the antibody–antigen interface were generated. Mutations were made to nearly every antigen-contacting residue using suggestions from computational methods. The most promising mutations were combined into a quadruple mutant with two mutations in the light chain and two in the heavy chain, and its crystal structure confirmed the predicted interactions.

A similar approach that focused on electrostatic interactions was employed to design single mutant variants with improved affinity. Selection criteria based on calculations of the improved binding electrostatics resulted in a success rate for single mutations of over 60%. By combining multiple designed mutations, the affinity of antibodies specific for various antigens was improved 10-fold for the anti-epidermal growth factor receptor antibody, cetuximab, and 140-fold for an anti-lysozyme antibody D44.1, achieving 52 and 30 pM affinity, respectively [167,168].

While antigen-contacting residues at the center of the binding interface may be an intuitive choice for mutations, many studies indicate that targeting peripheral residues may be more promising for affinity maturation. The key residues at the center of binding sites are usually hydrophobic and tightly packed and already well optimized for specific antigen interactions. In contrast, the surrounding residues are often hydrophilic and solvent exposed. Incorporating charged residues at the periphery of the interface may improve long-range interactions.

In the following example, the design strategy was based on two assumptions: (1) Mutation positions should be at the periphery of the antibody–antigen interface, and (2) substitutions should be those that frequently occur during affinity maturation in vivo. To improve the affinity of the therapeutic mAbs trastuzumab and rituximab, in silico models for a series of mutants were generated using crystal structures of the complexes, Monte Carlo-simulated annealing, and molecular dynamics simulation [169]. Single mutations at each of the 60 CDR positions to the 20 common amino acids were ranked by the total calculated binding free energy. The top 11 mutants were tested experimentally and only two of them showed improved binding. Alternatively, when only amino acids with a high usage in the binding sites of matured antibodies were considered for mutations, the success rate was 60% to 70%.

One of the most striking findings in this study was that affinity-enhancing mutations tend to cluster around positions where in vivo somatic mutations often occur. It is known that somatic hypermutation does not occur randomly within immunoglobulin V genes but is preferentially targeted to certain nucleotide positions, termed hotspots [170]. This process mainly results in the introduction of mutations that are located at or very near A/G|G|T/C|A/T and TAA sequences [171,172]. The results of the study indicate that germline hotspot sequences may point to the mutation sites in the affinity maturation process.

The combination of in silico calculations and thermodynamic analysis proved to be an effective strategy to improve the affinity of an anti-MCP-1 mAb 11K2 [173]. Amino acid substitutions were evaluated in each of the 62 CDR positions of 11K2, and all 20 amino acids were employed. Based on the crystal structure of 11K2 in complex with MCP-1, a virtual library of mutations to identify antibody variants of potentially higher affinity was generated. Each model of the mutated antibody–antigen complex was optimized by a combination of simulated annealing and molecular mechanics minimization. The variants were ranked by their electrostatic and van der Waals interaction energies and the most promising candidates were tested in vitro. Only mutations in the light chain of the antibody were effective at enhancing its affinity, suggesting that in this case, the interaction surface of the HC is not amenable to optimization. The single mutation with the highest affinity, N31R in CDR L1, yielded a variant with a five-fold higher affinity with respect to that of the wild-type antibody.

All these studies are examples of the fixed-backbone approach of computational design, where the backbones are not altered beyond energy minimization. Incorporating backbone flexibility in computational design allows conformational adjustments that may broaden the range of predicted low-energy sequences. In some cases, backbone movements are critical, for instance, when dealing with allosteric effects resulting from the changes in non-contacting residues. A comparison of different protocols for modeling backbone flexibility was performed in the affinity maturation study of the therapeutic mAb, trastuzumab [174]. An in silico approach based on the crystal structure of the trastuzumab complex with its target human epidermal growth factor receptor 2 (HER2) identified a key mutation D98W, which led to a three-fold affinity improvement of the already subnanomolar antibody.

Although the amino acid composition of protein–protein interfaces is quite diverse, there is a significant bias toward specific residues [175]. It was demonstrated that high-affinity antibodies could be obtained from restricted combinatorial libraries in which CDR positions are diversified to a combination of as few as two amino acids, Tyr and Ser [176]. Encouraged by these results, Inoue et al. [176] applied this binary code to the affinity maturation of an anti-lysozyme camelid single-domain antibody. They also used in silico screening for the selection of potential amino acid replacements. The scoring function was based on the interaction energy (IE) and electrostatic complementarity (EC) criteria [177]. When introducing mutations into CDR1 and CDR2, conserved amino acids were preserved, so that only about half of the residues were mutated to Tyr or Ser, giving a total of 512 (2^9^) theoretically possible mutants. Several variants that showed improved IE and EC parameters were tested for binding. The best of them exhibited a five-fold improvement in K_D_ values from 2.8 to 0.5 µM. Then, based on the crystal structure of the antibody–antigen complex, two residues in CDR2 in contact with the Ag were mutated to either Arg or Asp and tested for binding. This round yielded a variant with a K_D_ value of 0.14 µM, i.e., 20 times lower than in the parent mAb.

The design of mutants with improved affinities relies on the 3-D structure of antibody–antigen complexes. A variety of structure-modeling tools can help in the absence of experimental data. The following examples emphasize the value of computer modeling for affinity maturation of antibodies. They emphasize the use of computational docking, the process of predicting the conformation of a complex from its separated components.

In the first example, the binding of two antibodies to the stalk region of influenza hemagglutinin was modeled by using only the structure of the target protein and compared to the known experimental structures of the complexes [178]. This study demonstrated that some of the computational docking predictions can be very accurate, but the algorithm often fails to discriminate them from inaccurate solutions.

In a second example, the binding affinity of an anti-hepatitis B virus antibody was improved. For this study, both the antibody itself and the antibody–antigen complex were modeled (by docking the 17-residue peptide) [179]. Inspection of the model instructed the design of point mutations in the putative paratope, and two mutations, Y96S in VL and D98S in VH, were predicted to have the largest drop in free energy. The double mutant indeed exhibited a 10-fold increase of affinity.

While in silico design of antibody mutants may be successful, combinatorial libraries provide a common method to improve affinity. The selection of positions for diversification and the choice of amino acids for mutations are two principal tasks in a library design. As evidenced in the literature, the best results may be achieved if these tasks are fulfilled by a structure-guided approach.

A very convincing example of a stepwise structure-guided affinity maturation procedure was reported for anti-gastrin scFv TA4 [180,181]. An impressive 454-fold improvement in affinity was obtained by a combination of walking randomization [182] and a model-based approach that was achieved without experimental 3-D information. A structural model of the antibody–antigen complex was generated by docking a seven-residue peptide representing a linear epitope into the model of the antibody. The docked complex was refined by molecular dynamics, which indicated that the peptide adopts a helical conformation. Based on this model, four positions in CDR-H3 and five positions in CDR L3 were selected for randomization. The first of the libraries, that based on CDR-H3, produced a double mutant with an almost 10-fold improved affinity. The second library based on this mutant and CDR L3 mutagenesis produced two variants with about a two-fold affinity improvement over the double mutant. Again, the 3-D model guided the selection of positions in other CDRs for further affinity improvement. This procedure yielded several variants, with the affinity in the low nanomolar range.

Analysis of the binding surface of the antibody and assessment of the relative involvement of CDRs in target binding facilitates the strategy for library design. Depending on the CDR length and the number of interactions with the antigen, central CDRs H3 and L3 may provide the best opportunities for affinity maturation. This was the case in the development of an anti-VEGF scFv isolated from a phage-displayed human antibody repertoire [183]. Two phage display libraries were constructed by diversification of CDR-H3 and CDR L3. A competitive phage-selection strategy in the presence of the parental scFv as a competitor was used to eliminate low-affinity binders. High-affinity variants were retrieved from both libraries. An optimized VL variant was designed and constructed by combining recurrent replacements found among selected variants into a single molecule, resulting in an additional affinity increase. Further affinity improvements were achieved by combining this optimized VL with the best VH variants. The final variant showed an 18-fold affinity improvement over the parental scFv and exhibited an enhanced potency to block the binding of VEGF to its receptor.

An impressive example of affinity improvement was carried out for the anti-complement protein receptors C5aR1/2 mAb [184]. The affinity of the parental antibody was improved by randomizing amino acids in CDR-H3 and CDR L3 using a phage library displaying scFv fragments. Following recombination of the two libraries and screening to identify additional synergistic increases in affinity, the best variant was selected with four mutations in CDR-H3 and two mutations in CDR L3. This variant binds its target with an affinity in the low pM range, demonstrating a gain of three orders of magnitude with respect to the parental antibody affinity.

Quite often, central CDRs, particularly CDR-H3, are too heavily involved in the interactions with the antigen, so that CDR-H2, and to a lesser extent CDRs H1 and L1, may be the focus of library design. Increasing the number of diversified positions in each library and expanding the selection to all CDRs inevitably results in a lower coverage. However, in certain cases, this approach may also be successful. Simultaneous mutagenesis of all six CDRs in a non-human primate antibody that neutralizes anthrax toxin was carried out using phage display technology [185]. The library contained 5 × 10^8^ variants, with each variant containing an average of four mutations. The best variant selected from the library showed a 19-fold affinity improvement to 180 pM.

While selecting the positions for mutagenesis to improve binding, one may consider not only the paratope residues or surrounding residues in the Vernier zone but also positions in the core of the variable domains or even at the elbow between the variable and constant domains. Exactly this approach was realized during the development of the anti-tumor growth factor beta subunit 1 (TGF-β1) antibody metelimumab [186]. Upon conversion from the parental single-chain variable fragment (scFv) to IgG4, the binding affinity dropped by 50-fold. Following a hypothesis that this was due to decreased conformational flexibility of the IgG, insertion mutants in the elbow region were designed and screened for binding and potency. The insertion of two glycines in both the heavy chain and light chain elbow regions restored the binding affinity. The crystal structure of the mutant confirmed that the insertions provided enough flexibility for the variable domains to extend further apart than in the wild-type Fab, allowing the CDRs to make additional interactions not seen in the wild-type Fab structure.

### 2.4. Specificity

Conventional antibodies are monospecific and typically recognize a single antigen exclusively, owing to the binding of non-linear epitopes. Some antibodies do exhibit multi-specificity, particularly if a very similar epitope is present on more than one antigen. Typical examples include species cross-reactive antibodies that recognize orthologous proteins in different species [187,188] or antibodies that interact with different members of a conserved protein family [189,190]. The species specificity has often been a setback in assessing antibody utility as a therapeutic agent in various animal models. The same combinatorial techniques that are used to improve antibody affinity have been used to modify their cross-reactivity [191,192]. Specificity engineering also heavily relies on in silico design strategies and the availability of experimental structural information [193].

Using the crystal structure and molecular mechanics-based energy function, cross-species specificity was introduced into the antibody that inhibits cancer-associated serine protease MT-SP1 [194]. The mAb exhibits a K_D_ value of 12 pM towards human antigen but only 4 nM towards the mouse ortholog. There are only three residues on the protease surface that both make contact with the antibody and that are different between the human and mouse versions of the enzyme, but these residues are not critical for inhibition. Computational design was used to predict a suite of mutations that could improve the affinity to the mouse antigen. Mutations were introduced at six positions within 5 Å from these three epitope residues. Each of the selected residues, two in CDR-H1 and four in CDR-H3, was mutated in silico to all possible amino acids, and for each substitution, the change in binding energy was calculated. Most of the mutations were predicted to be neutral. Out of eight candidates tested experimentally, one variant, T98R, improved the affinity by an order of magnitude without any effect on the binding of the human ortholog.

The development of a promising therapeutic mAb targeting quiescin sulfhydryl oxidase-1 (QSOX1) was hampered by the lack of reactivity against the mouse QSOX1 ortholog [195]. To understand the molecular basis for species restriction, the crystal structures of mouse QSOX1 alone and human QSOX1 in complex with the Fab fragment were determined. Structural differences responsible for the species specificity of the antibody were identified and used for the construction of small libraries, in which up to four positions near key epitope positions were diversified. After several rounds of panning and the combination of mutations from different libraries, the affinity toward mouse QSOX1 was improved by at least four orders of magnitude, reaching the low nanomolar range and matching the affinity toward human QSOX1. The crystal structure of the re-engineered variant complexed with its mouse antigen revealed that the antibody accomplished dual-species targeting through altered VH–VL domain orientation and, most importantly, through rearrangement of the CDR-H3 backbone because of a quadruple mutation YYGS to SMDP.

In another study, a structure-based strategy was implemented to develop an anti-CD81 mAb to enable animal model testing in cynomolgus monkeys [196]. The antibody would bind tightly to human CD81 (K_D_ vakue of 0.9 nM) but exhibited no detectable binding to cynomolgus (cyno) CD81. The crystal structure of the scFv was determined in complex with human CD81 and used for guiding the library design. A phage-display library was constructed to diversify CDR-H2, which seemed to be a major specificity determinant. Alternating rounds of binding selections with immobilized cyno and human antigens yielded an antibody that was used as a template for the second round of selection with libraries around CDR L1 and CDR-H1. The best variant exhibited robust binding to cyno CD81 and only showed a two-fold reduction in affinity for human CD81.

Whereas species cross-reactivity or broad neutralizing potential may be beneficial for a therapeutic antibody, sometimes, the insufficient selectivity may be regarded as a liability. Although mAbs are generally very selective, close analogs of the target molecule may pose a risk of side effects. The development of anti-progesterone mAb C12G11 was hampered by its poor selectivity [197]. The mAb has a picomolar affinity for progesterone but also strongly binds 5β- and 5α-dihydroprogesterone. To reduce the cross-reaction with these analogs, a phage library randomizing five antigen-binding positions in CDR-H2 and CDR L3 was constructed. The design was based on the homology model of the antibody complemented by docking of the target molecules. Variants selected in the initial screening were further optimized by the addition of second-sphere positions to the library. The best variant demonstrates high specificity toward progesterone as compared with the 15- to 20-fold lower cross-reactivity for the analogs. The improvements are linked to a change in the canonical class of CDR L3.

Koenig et al. faced a similar task to fine-tune the specificity of an angiopoietin-2 (Ang2)/VEGF dual-action Fab [198]. This antibody utilizes overlapping CDR sites for dual antigen interaction, with affinities in the sub-nanomolar range. However, it also exhibits significant (K_D_ value of 4 nM) binding to Ang1, which has high sequence similarity to Ang2. An approach to specificity engineering that does not require prior knowledge of the antibody–antigen interaction was employed in this study. A large phage-displayed library of the Fab variants with all possible single mutations in all six CDRs provided information on the effect of binding for each mutation. In silico analysis identified 35 mutations predicted to decrease the affinity for Ang1 while maintaining the affinity for Ang2 and VEGF. Structural analysis showed that some of the mutations cluster near a potential Ang1/2 specificity-determining residue, while others are up to 15 Å away from the antigen-binding site and apparently influence the binding interaction indirectly. The lack of information on antibody–antigen interactions in this approach was compensated for by the size of the library.

The mechanisms of antigen recognition by antibodies vary significantly from the structurally rigid key-and-lock mechanism to the adaptable induced-fit mechanism. Correspondingly, one of the mechanisms of multispecificity lies in the plasticity of the antigen-binding site, which allows for the recognition of structurally unrelated epitopes by the same antibody [199,200]. This principle was utilized in a stepwise engineering strategy for generating dual-specific antibodies de novo, called two-in-one antibody with dual-action Fab (DAF). The first proof-of-concept DAF was targeting VEGF and HER2 [201,202]. The strategy was also successfully applied for the generation of duligotuzumab, which targets EGFR and HER3 [203].

### 2.5. Chemistry, Manufacturing, and Control (CMC) Considerations

Recombinant mammalian cells are the dominant production system for antibody-based therapeutics because of their ability to perform complex post-translational modifications (PTMs) that are often required for efficient secretion, drug efficacy, and stability [204]. Because of the nature of heterologous expression, there are modifications to the biologics, which include misfolding and aggregation, oxidation of methionine, deamidation of asparagine and glutamine, variable glycosylation, and proteolysis (see Table 2) [205,206]. Such unintended PTMs can pose challenges for consistent bioprocessing and can affect the molecular physicochemical properties (such as shape, size, hydrophobicity, and charge) that in turn can affect pharmacokinetic (PK) and pharmacodynamics (PD) properties. For instance, electrostatic interactions between anionic cell membranes and the predominantly positive surface charge of most antibodies can influence the blood concentration and tissue disposition kinetics in a manner that is independent of antigen recognition. Thus, charge variation can result in shifts in isoelectric point values, which can change the tissue distribution and kinetics; increases in net positive charge generally result in increased tissue retention and increased blood clearance [207,208,209]. Protein and peptide deamidation can occur spontaneously in vitro under relatively mild conditions that can be used to predict in vivo chemistries [210]. For antibodies and other therapeutic proteins, great effort is placed in the manufacturing process and storage conditions to minimize this form of degradation. Glycosylation control is typically controlled by the selection of the manufacturing cell line and control of cell metabolism during bioreactor conditions [211,212]. The specific glycan attachments (fucosylation, sialylation, galactosylation, high-mannose, and bisecting glycans) have great importance to the antibody properties [213,214] (See Figure 5). Simulations of the dynamic interface between the glycans and the Fc domains have been described [215]. However, it is also important to remember that under physiological temperature and pH conditions, antibody deamidation, c terminal cleavage, and glycation kinetics do occur and can affect the serum lifetime of antibodies [209,216,217].

During the process of discovery, the selection of candidate molecules should employ technologies that increase the odds of identifying potent biologics that bind the desired biologically relevant epitope. Concomitant with potency optimization, the biologics should be counter-screened to have drug-like properties early in the process. Although the germline amino acid sequences are typically left unchanged, mutagenesis of amino acids’ liabilities (sites of oxidation, deamidation, clipping, glycation, glycosylation) in the CDRs should be considered. Such changes should be completed prior to the manufacturing cell line development to minimize the risks of having a less-than-robust chemistry, manufacturing, and control (CMC) process. Upstream consideration of developability metrics should reduce the frequency of failures in later downstream development stages. Optimization of stability in the early stages of discovery can reduce complications in upstream and downstream process optimization as well as increase the potential for successful drug product formulations [218,219]. Nonetheless, downstream processing can minimize some levels of oxidation via the presence of free radical scavengers, elimination of redox metal ions, addition of chelation agents, protection from light, decrease in storage temperature, and reduction of exposure to oxygen.

#### 2.5.1. Solubility

Low solubility or high viscosity of antibody formulations at concentrations over 100 mg/mL can impede their development as products suitable for subcutaneous delivery. Antibody engineering, especially when applied at the discovery stage, may be instrumental in overcoming the challenges of the product development and pave the path to the clinic. The following examples highlight some approaches that proved to be helpful in improving the solubility of antibodies. Several studies were based on homology modeling rather than on experimental crystal structures, and in many cases, this may be enough for developability purposes. Of course, this excludes the cases when antibodies present unusual CDR in either HC or LC loops either in sequence or in length. Antigen-bound co-crystals are also very useful for identifying mutants that are more likely to retain target binding while optimizing solubility.

Structure-guided design of point mutations was carried out for the development of a therapeutic mAb candidate that was unacceptably viscous at high concentrations [270]. The idea was to test the effects of hydrophobic and electrostatic intermolecular interactions on the solution behavior of the mAb by disrupting either aggregation prone regions or clusters of charged residues. The variable region contained two hydrophobic surface patches and a negatively charged cluster. The disruption of a hydrophobic patch at the interface of VH and VL via L46K mutation in VL destabilized the mAb and abolished antigen binding. However, mutation at the preceding residue (V45K) in the same patch increased the apparent solubility and reduced viscosity without sacrificing antigen binding or thermal stability. Neutralizing the negatively charged surface patch by E60Y mutation in VL also increased apparent solubility and reduced viscosity of the mAb, whereas charge reversal at the same position (E60K/R) caused destabilization, decreased solubility, and led to difficulties in sample manipulation that precluded their viscosity measurements at high concentrations. Both V45K and E60Y mutations showed similar increases in apparent solubility. However, the viscosity profile of E60Y was considerably better than that of the V45K, providing evidence that intermolecular interactions in this mAb are electrostatically driven.

Aggregation of single-domain VH antibodies specific for Alzheimer’s amyloid β-peptide was examined from the structural perspective [271,272,273]. These antibodies contained clusters of hydrophobic residues within the HC and LC CDR3. Inserting two or more negative charges at each edge of the CDR3 domains potently suppressed antibody aggregation without altering binding affinity. Inserting charged mutations at one edge of CDR3, either the N- or C-terminal, also prevented aggregation, but only if such mutations were located at the edge closest to the most hydrophobic portion of CDR3. In contrast, charged mutations outside of CDR3 failed to suppress aggregation. These findings demonstrate that the CDR loops can be engineered in a systematic manner to improve antibody solubility without altering binding affinity.

The case of anti-IL-13 mAb CNTO607 presents three structure-based engineering approaches to improved the solubility of a therapeutic candidate [274]. First, the isoelectric point was modified by the incorporation of charged residues in the positions remote from the binding site. A mutant with a modified pI showed a two-fold improvement in solubility while retaining full binding to IL-13. Second, the overall surface hydrophobicity was targeted by mutating residues in a hydrophobic patch found in CDR-H3. According to the crystal structure, the patch included residues F99-H100-W100a from CDR-H3 flanked by hydrophobic residues from the light chain. The triad in CDR-H3 appeared to be essential for the high affinity of the antibody to IL-13. Various mutations in these residues improved solubility but negatively impacted affinity. Mutations in CDR L3 were more promising since this CDR is less involved in antigen binding. The best variant with W91Y and M93S mutations gained a two-fold improvement in solubility and exhibited a shorter retention time on HIC, while binding to IL-13 remained in the low picomolar range. In a third approach, an N-linked glycosylation site was introduced in CDR-H2 (D53N) to shield the aggregation patch in CDR-H3. This variant indeed showed greatly improved solubility while maintaining affinity to IL-13 and proved to be the most effective route for enhancing the solubility of CNTO607. Recently, there has been a description of how optimization of the V domain framework can improve the biophysical qualities of a therapeutic antibody candidate [275].

Another example of solubility improvement through computer modeling was reported for an integrin α11-binding antibody [276]. A homology model of the parental Fv region revealed hydrophobic patches on the antigen-binding surface. A series of 97 computationally designed variants focused on the residues in the hydrophobic patches that were expressed, and their HIC retention times were measured. As intended, many of the variants reduced the overall hydrophobicity as compared to the parental antibody. Contrary to the previous study (CNTO607), replacement of aromatic residues W96, Y97, and Y98 in CDR-H3 did not cause a loss of binding, apparently because they are not in contact with integrin. Interestingly, adding charged residues in place of polar residues in the CDRs near the hydrophobic patches did not reduce the retention time.

Three-dimensional protein property descriptors were developed and evaluated for their ability to predict the hydrophobicity profiles of antibodies [276]. Analysis of recently published data for 137 clinical mAb candidates [277] indicated that the surface area of hydrophobic patches consistently correlated to the experimental HIC data across a diverse set of biotherapeutics.

A general approach to predicting aggregation-prone regions on the basis of three-dimensional structures has been realized in the algorithm termed AggScore [278]. The method uses the distribution of hydrophobic and electrostatic patches on the surface of the protein, factoring in the intensity and relative orientation of the respective surface patches into an aggregation propensity function that has been trained on a benchmark set of 31 adnectin proteins. When applied to the experimentally characterized antibodies in the clinical stage [277], AggScore accurately identified aggregation-prone regions and predicted changes in aggregation behavior upon residue mutation.

As more biotherapeutics are entering pharmaceutical pipelines, more weight is put on the early-stage developability assessment and optimization strategies. Computational methods for assessing solubility, hydrophobic interactions, and other liabilities are in high demand. Successful efforts have also been made to use rational design to reduce aggregation and improve solubility by mutating key surface residues identified from a crystal structure or a homology model.

#### 2.5.2. Stability

After the selection based on functional properties, antibodies may be modified to improve developability or scale-up processes involving stability, expression, purification, and formulation. The stability of an antibody is influenced by a number of factors that can include: (1) core packing of individual domains that affects their intrinsic stability; (2) protein–protein interface interactions that have impact upon the HC and LC pairing; (3) burial of polar and charged residues; (4) hydrogen-bonding network for polar and charged residues; and (5) surface charge and polar residue distribution among other intra- and inter-molecular forces [279]. Potential structure-destabilizing residues may be identified based upon the crystal structure of the antibody or by molecular modeling in certain cases, and the effect of the residues on antibody stability may be tested by generating and evaluating variants harboring mutations in the identified residues. One of the ways to increase antibody stability is to raise the thermal transition midpoint (T_m_) as measured by differential scanning calorimetry (DSC), differential scanning fluorimetry (DSF), or thermal transitions [280,281,282,283,284]. In general, the protein T_m_ is correlated with its stability and inversely correlated with its susceptibility to unfolding and denaturation in solution and the degradation processes that depend on the tendency of the protein to unfold [285]. A few studies have found a correlation between the ranking of the physical stability of formulations measured as thermal stability by DSC and physical stability measured by other methods [169,286,287,288,289]. Formulation studies suggest that a Fab T_m_ value can have implications for the long-term physical stability of a corresponding mAb. Thus, the CDR sequence selection can impact the stability of the VH–VL domain, and sequence–stability tradeoffs must be considered during the design of such libraries [290].

## 3. Engineering Antibody Activity

While mAbs are successful for many distinct applications, there are still limitations. First, the surface area of the IgG variable region may not bind to the small extracellular loops of transmembrane proteins, such as G-protein-coupled receptors (GPCRs). Secondly, animal models show that most administered mAbs have limited distribution into the diseased tissue. A favorable pharmacokinetic profile does require sufficient target occupancy in the diseased tissue, which ultimately requires efficient tissue penetration and retention time in the diseased tissue. Thirdly, single agent mAb efficacy can be limited because the disease phenotype can have more than one pathway that can mediate resistance. Often, the in vitro properties of the candidate antibody that probe a limited array of responses do not corroborate with in vivo profiles that can involve more complex mechanisms of action. These limitations have prompted research in generating new antibody-based therapeutics that can meet these aforementioned challenges by adopting antibody engineering approaches that can include: Binding domain engineering; avidity modulation; antibody–drug conjugation; Fc activity engineering; and bispecific antibody generation.

### 3.1. Binding Domain Engineering

The average surface area of an antibody epitope-containing surface is around 1600 to 2300 Å^2^ [291]. Although this surface area is ideal for modulation of most protein–protein interactions, there can be target molecules with epitope surface exposures that are more restricted. For instance, there are limited examples of the obtainment of functional antibodies against GPCRs. Recent crystal structures have shown that there is limited access to epitopes due to the presence of N-terminal domain glycosylation and limited surface exposure to the extracellular membrane protein loops [292]. For applications that require smaller binding surfaces, single domain (12–15 kDa) antibodies (sdAbs, also known as VHH Abs or nanobodies) can be more suitable as targeting proteins. For such applications, VHH Abs, derived from camelid family heavy chain Abs, have smaller binding surfaces that could bind to smaller cryptic regions of GPCRs. These single domain binding proteins show promise for stabilizing active GPCR conformations and serve as chaperones for co-crystallization [293,294,295,296,297]. The VHH domain structure lacks the human or mouse mAb HC-LC structure (which are hydrophobic at their pairing interface), resulting in a surface that is much more hydrophilic than that of an IgG Fab region [298,299]. Therefore, camelid-derived VHH nanobodies tend to have favorable biophysical characteristics, like high solubility and low aggregation, compared to human sdAbs [300]. The smaller molecular weight can expand the range of drug-dosing modalities to include inhalation, needle free, oral, topical, and ocular delivery [298,299,301,302].

In addition, the VHH CDR3 is often longer than the IgG VH CDR3, potentially allowing it to form more favorable contacts with its binding epitope [300]. Recently, Caplacizumab (ALX-0081), an anti-von Willebrand factor humanized VHH, was launched in 2018 for the treatment of thrombotic thrombocytopenic purpura and thrombosis [303]. Since in silico analysis showed that camelid VHH sequences could be aligned to human IGKV and IGLV families based on canonical structure and sequence homology, optimization of primary sequence is possible to minimize the potential of the development of anti-drug antibodies (ADAs) [304].

Besides camelids, cartilaginous fishes produce a distinct heavy chain Ab subtype containing a single variable region immunoglobulin new antigen receptors (VNARs), These 11- to 14-kDa domains comprise two heavy chains with an antigen-binding region with no associated light chains [305] and can bind with high affinity and specificity to target molecules [306,307]. These molecules have 8 beta strands instead of the 10 beta strands found in VHH and mammalian IgGs. In addition, VNARs lack the CDR2 domains and longer hinge regions found in mammalian IgG molecules. Thus, the diversity of VNARs depends primarily on the CDR3 domain. With their small size and single domain format, VNARs are highly stable and can be produced at high levels using different expression systems [308]. Because of their size, there are recognition niches that are unique for such therapeutic sdAbs [309,310,311]. The development of libraries for selection has expanded the utility of generating potent molecules [312,313]. As with nanobodies, these molecules can be engineered to be more human-like and have been used to isolate binders to CXCR4, a druggable GPCR [314,315], HER2, PD1, and glypican 3 [316].

### 3.2. Avidity Modulation

Because each antibody has two antigen-binding sites, antibody engagement to the antigen can be multivalent when there is more than one antigen on the target surface [317]. For avidity to occur, the antigen sites must be present at a sufficient density, such that once the first Fab has bound, the second Fab can bind before the first Fab dissociates. Thus, the nature of an IgG engagement to the antigen can be more complicated than a single binding event. Rather, the functional affinity or avidity represents the accumulated strength of multiple affinities to an antigen [318,319]. In such cases, the Fab region can modulate protein–protein interactions of the antigen. Occasionally, the structural nature of the Fab region–epitope engagement can limit target neutralization via constrained avidity through steric occlusion [320]. The pharmacokinetic profile of the molecule should allow for sufficient time to allow for kinetics of avidity to occur. If not, there will be an apparent loss of potency even though there may be steep saturation curves from in vitro experiments [321].

However, Fc region clustering due to FcγR interactions can contribute to Fab target avidity [322,323,324]. The avidity due to Fc region–FcγR crosslinking can affect the immune cell effector function that can contribute to autoimmune disorders [325,326,327,328,329]. In such indications, effective FcγR blockade requires doses of intravenous immunoglobulin. However, this approach requires careful consideration of potential safety concerns related to the induction of serious acute events, such as cytokine release, platelet activation/aggregation, and complement activation [330].

Monoclonal antibodies that target the inhibitory immune checkpoint receptors, such as CTLA-4 and PD-1, stimulate antitumor immunity to treat advanced melanoma, lung cancer, and other types of human cancer [331,332]. Such agonist antibodies against the immunostimulatory receptors on T cells and antigen-presenting cells were designed to have more silent Fc regions to prevent Fc effector function but retain Fab region avidity to stimulate antitumor immunity [333]. Immune effector cells have stimulatory receptors belonging to the tumor necrosis factor (TNF) receptor superfamily (such as OX40, CD27, 4-1BB, and GITR) [334,335]. There has been much effort to develop the use of their respective ligands and agonist antibodies to activate these receptors to stimulate the proliferation and activation of T cells [336,337,338,339] to mediate anti-tumor activities [340,341,342].

Agonistic activities of immunomodulatory antibodies require the engagement of different types of Fc receptors and cell surface receptors. To activate downstream signaling pathways, TNF receptors undergo higher-order clustering upon binding to their respective trimeric ligands [343]. Thus, regular antibody binding may not be enough to induce the required threshold TNF receptor clustering that can occur with the binding of trimeric ligands. Instead, antibody crosslinking via Fc engagement is necessary for receptor activation in in vitro assays [344,345,346,347]. The crosslinking of IgG Fc to FcγRIIB receptors can multimerize more than one antibody molecule, which in turn can facilitate the clustering of enough TNFR for signaling pathway activation. Recent studies in mice indicated that the engagement to the inhibitory FcγRIIB receptor is critical for the agonistic activity of antibodies to a number of TNFR targets, such as CD40 [340,348], death receptor 5 (DR5) [340,349], and CD95 [350]. If such antibodies have Fc regions that can engage various activating FcγRs, effector functions, such as ADCC and ADCP, can be induced and deplete these targeted immune cells. Nonetheless, the anti-OX40 and anti-GITR antibodies may facilitate the selective elimination of intratumoral regulatory T cells in the tumor microenvironment by the effector functions of the antibody [351,352]. Such antibody-mediated killing of regulatory T cells may be more important than the antibody-mediated activation of effector T cells for the anti-tumor activities of therapeutic anti-OX40 and anti-GITR antibodies.

By design, human IgG antibodies have low binding affinities to most human Fc receptors except FcγRI [353]. To optimize the anti-tumor activity of agonist antibodies’ binding to immunostimulatory TNF receptors, the Fc region of the IgG antibody was engineered to bind more strongly to the FcγRIIB receptor. In particular, Chu et al. introduced the S267E/L328F mutations on an anti-CD19 IgG1 Fc to enhance FcγRIIB-binding affinity that resulted in improved inhibition of B cell receptor-mediated activation of primary human B cells [354]. However, this Fc variant also enhanced binding to the R131 allotype of the activating FcγRIIA receptor [355]. Mimoto et al. utilized the V12 mutations (E233D/G237D/P238D/H268D/P271G/A330R) in the IgG1 Fc to selectively enhance FcγRIIB engagement without an associated increased binding to either the H131 or R131 allotype of the FcγRIIA receptor [356]. Mutations that abrogate FcγRIIIA binding can decrease the potential ADCC activity. An anti-CD137 agonistic antibody with the V12 mutations showed enhanced agonistic activity dependent on FcγRIIB engagement with less ADCC activity that was linked to FcγRIIA binding. Alternatively, FcγRII-binding Centyrins can be fused to therapeutic antibodies to bind to FcγRIIB receptor (FcγRIIB), thereby enabling the antibody multimerization that drives TNFR activation [357].

Ab agonistic activity depending on FcγRIIB engagement depends on the FcγR expression in the local microenvironment. To augment the agonism of immunostimulatory antibodies independent of FcγR engagement, White et al. recently reported that human IgG2 subtype can impart super-agonistic activity to immunostimulatory antibodies for CD40, 4-1BB, and CD28 receptors [358]. This activity is conferred by a unique configuration of disulfide bonds in the hinge region of the IgG2 subtype and is not dependent on FcγRIIB engagement. To add to the repertoire of Fc mutations that can promote antibody multimerization without the need of FcγRIIB crosslinking, Diebolder et al. reported that selective Fc mutations can facilitate hexamerization of IgG Abs upon binding targets on the cell surface [359]. Specific noncovalent interactions between Fc regions resulted in the formation of ordered antibody hexamers after antigen binding on cells. These hexamers recruit and activate C1q, the first component of complement, to trigger the complement cascade. The interactions between neighboring Fc segments could be manipulated to block, reconstitute, and enhance complement activation and killing of target cells, using all four human IgG subclasses [360]. In contrast, the E345R mutation on an anti-OX40 antibody had increased agonism by promoting the clustering of OX40 receptors without the dependence on FcγRIIB cross-linking [361]. This cross-linking to FcγRIIB can lead to a further boost of the agonism of the anti-OX40 antibody with an IgG1 Fc but not with the silent IgG2σ Fc region, which lacks binding to FcγRs. The ADCC and CDC activities of the anti-OX40 antibody with the E345R mutation were affected by the choice of IgG subtypes [362]. With so many oligomeric Ab targets, there are continuing applications of hexameric therapeutic Abs that can affect downstream signaling events [328,329,363].

Alternatively, when an IgG format does not achieve enough of an effect on a cell surface receptor, the variable regions can be transferred to an IgM format to elicit the functional activity through avidity. Clearly, the IgM’s higher valency can facilitate receptor crosslinking. For instance, when anti-trail-receptor IgG did not elicit a strong response, the switch to an IgM format resulted in stronger induction of trail-receptor-induced apoptosis [364]. Antibody formats that promote crosslinking are being assessed in clinical trials [365]. Likewise, other hinge and isotype formats also affect binding to targets [366].

### 3.3. Antibody–Drug Conjugates

The high binding specificity of antibodies can be combined with the potent cytotoxicity of small molecule agents to generate targeted therapies with higher therapeutic indices than traditional chemotherapeutics. By delivering toxic payloads only to cells that express specified antigens, it is possible to confine toxicity to malignant tissue while theoretically minimizing collateral damage. Antibody–drug conjugates (ADCs) bearing cytotoxic moieties should ideally target antigens that are present at significantly higher amounts on tumor cells. For many ADCs, it is also beneficial to bind to internalizing receptors, which deliver the conjugate into the cell and allow the active moiety to elicit its effects.

Many types of cytotoxic agents can be conjugated to antibodies for concentration into target cells. Among these, the most common are natural products, such as the maytansinoids (derived from the macrolide maytansine of *Maytenus* plants), auristatins (derived from Dolastatin peptides of *Dolabella auricularia* sea hares), and calicheamicins (enediyne antibiotics from *Micromonospora echinospora* bacteria). The auristatins, exemplified by monomethyl auristatins E and F (MMAE, MMAF), and the maytansinoids, including DM1 and DM4, are microtubule inhibitors while calicheamicins like γ1 act by creating double-stranded DNA breaks. Due to the remarkable cytotoxicity of these agents, they must be tethered to antibodies for targeted delivery and reduction of systemic toxicity.

It was earlier recognized that the sub-picomolar potency of calicheamicins would allow for efficient tumor killing when coupled to antibodies for specific delivery. In 1993, Hinman et al. reported ADCs combining calicheamicins γ1, α2, α3, N-acetyl-γ1, or pseudoaglycone (PSAG) with a monoclonal antibody against the internalizing antigen, polyepithelial mucin [367]. Hydrazide analogs of the calicheamicins were prepared and conjugated to oxidized glycan residues on the antibody. A comparison of conjugate analogs revealed the importance of the rhamnose sugar in the DNA-binding region of the drug, whereas a distal amino sugar residue was more amenable to substitution or removal. Stabilization of the linker with the addition of disulfide-proximal methyl groups served to increase the therapeutic index of the ADCs.

Such calicheamicin-loaded ADCs have proven effective for the treatment of leukemia. The first approved ADC was gemtuzumab ozogamicin, which demonstrated an ablation of acute myeloid leukemia (AML) cells [368]. To form the ADC, gemtuzumab (anti-CD33) is linked to N-acetyl-γ-calicheamicin dimethyl hydrazide via non-specific lysine conjugation and a 4-(4-acetylphenoxy) butanoic acid spacer. The average drug-to-antibody ratio (DAR) is two to three, although some individual antibodies remain unconjugated and others have higher DAR values. After its approval in 2000, the ADC was voluntarily withdrawn in 2010 due to concerns over its toxicity and lack of efficacy. In 2017, the drug was re-approved after a meta-analysis and new clinical data indicated a benefit for the treatment of AML (history reviewed in [369]). Meanwhile, inotuzumab ozogamicin, a CD22 antibody conjugated to the same linker-drug moiety, demonstrated cytotoxicity against B cell lymphomas and was approved for treatment of acute lymphoblastic leukemia (ALL) in 2017 [370,371]. Clearly, calicheamicins possess satisfactory potency that, when combined with antibody specificity, allows for successful elimination of hematological malignancies.

Auristatins have also been conjugated to tumor-targeting antibodies to elicit specific and potent tumor killing. Doronina et al. attached auristatin analogs to antibodies targeting Lewis Y antigen and CD30 and compared the properties of ADCs containing acid-labile hydrazone linkers with those containing protease-sensitive dipeptide linkers [372,373]. Drug conjugation was more site specific in this case, using a maleimide group to form a covalent bond with reduced thiols from antibody cysteine residues. Since antibodies contain four relatively exposed inter-chain disulfide bonds, uniform drug loading of approximately eight auristatins per antibody was achieved. Peptide linkers, in particular, a valine–citrulline linker between the antibody and monomethyl auristatin E (MMAE), showed increased stability compared to more traditional hydrazone linkers. As a result, such linkers allowed for more specific delivery and lower systemic toxicity. Thus, optimization of conjugation and linker chemistry is important for maximization of the therapeutic index.

Subsequent work with CD30-MMAE antibodies explored the effect of drug loading on the therapeutic properties of ADCs [374]. By incubating antibodies with varying ratios of linker–drug and purifying different species using hydrophobic interaction chromatography, ADCs with defined DAR values of 2, 4, and 8 were generated. While in vitro ADC potency increased with increasing DAR, the in vivo activity was less dependent on drug loading. At equal doses, DAR 4 and DAR 8 ADCs demonstrated similar efficacy in vivo, whereas the DAR 2 species had slightly lower activity. Pharmacokinetic analysis revealed that the lower DAR species had longer half-lives and greater exposure, explaining why the DAR 8 species with high in vitro activity was not more effective in the mouse xenograft study. The intermediate DAR species progressed into clinical trials and was approved in 2011 as brentuximab vedotin for the treatment of Hodgkin lymphoma and systemic anaplastic large cell lymphoma [375].

The third main class of cytotoxic agents is the maytansinoids. In 1992, maytansine was derivatized with a disulfide group, which could be reduced and conjugated to disulfide or maleimide groups on a chemically modified HER2 antibody [376]. Chemical handles were added to the antibody via non-specific amine coupling and allowed for DAR values ranging from 1 to 6. The DAR 4 ADCs were shown to have maximal cytotoxicity in an in vitro assay. ADCs with the cleavable disulfide linker were significantly more potent than ADCs with a non-cleavable thioether linkage, presumably due to the more efficient release of the active drug within target cells. However, another study with auristatin ADCs demonstrated that non-cleavable linkers may have better therapeutic windows because of less non-specific drug release [9]. An important factor is the activity of the modified drug moiety that results from proteolytic digestion of ADCs with non-cleavable linkers.

Trastuzumab emtansine represents a successful combination of HER2 antibody, DM1 maytansinoid, and a non-cleavable linker. Lewis Phillips et al. compared trastuzumab-DM1 conjugates containing a panel of reducible disulfide linkers and a non-reducible linker [377]. The ADC with a non-cleavable linker based on thiol-maleimide conjugation to the maytansinoid and amine-succinimide conjugation to the antibody unexpectedly caused the greatest tumor inhibition in vivo. An increase in pharmacokinetic stability likely contributed to this effect, as the ADCs with reducible linkers were more likely to lose the payload before delivery to target cells. While the bystander effect does not occur when a non-cleavable linker is used due to the inability of the charged metabolite to cross membranes, lower systemic toxicity and full targeted delivery of cytotoxic payloads seem to make up for this defect. Trastuzumab-DM1 ADCs using this non-reducible linker progressed to clinical trials, and in 2013, they were approved for the treatment of HER2-positive breast cancer [378].

While the paradigm for successful ADCs has revolved around the most potent cytotoxic agents and stable linkers, other strategies have also been explored. Sacituzumab govitecan is an ADC targeting Trop-2 that contains the active metabolite SN-38 from the topoisomerase inhibitor irinotecan [379]. SN-38 is less toxic than traditional ADC payloads, having the half maximal inhibitory concentration (IC50) in the nanomolar, rather than picomolar, range. Thus, a higher DAR (7–8 compared to the more typical 3–4) is required to elicit sufficient tumor cytotoxicity. Additionally, the ADC makes use of a pH-sensitive carbonate linker, which releases drug in the lysosome of target cells but also into the circulation, with a half-life of approximately one day. This semi-stable linker is proposed to allow for the bystander effect by which molecules of SN-38 diffuse to neighboring tumor cells that may have a lower expression of Trop-2. While Trop-2 is expressed on a number of tumor types, sacituzumab govitecan has been most studied in cases of triple negative breast cancer.

In addition to natural products, toxins have also been conjugated or fused to antibodies to generate tumor-targeting immunotoxins. For example, Mansfield et al. described a disulfide-stabilized (HC R44C, LC G100C) Fv targeting CD22 that was fused to a 38-kDa truncated form of *Pseudomonas* endotoxin A (PE38) via the C-terminus of the VH domain [380,381]. The PE38 moiety contains translocation and ADP-ribosylating regions that allow it to inactivate elongation factor 2 within the cytosol of the target cell after delivery by the Fv domain. This activity leads to an inhibition of protein synthesis, induction of programmed cell death, and allowed for reduction in tumor growth in a mouse xenograft model. Subsequently, the CDR3-H3 of the Fv domain was mutated to improve CD22 binding while the PE38 domain was stabilized with the R490A mutation to reduce proteolysis [382]. The resulting molecule, moxetumomab pasudotox, was approved in 2018 for the treatment of hairy cell leukemia.

Radionuclides represent an additional class of payload that can be attached to antibodies to create antibody–radionuclide conjugates (ARCs). In one case, yttrium-90 was conjugated to the CD20 antibody ibritumomab to generate ARCs for the treatment of lymphoma [383]. The ^90^Y isotope was used due to its generation of beta particles, which penetrate several millimeters to elicit a bystander effect, and its favorable decay half-life of 2.7 days. Conjugation was achieved using the linker-chelator tiuxetan, whose isothiocyanate group forms a stable thiourea bond with antibody amines. Notably, the ^90^Y ARC was used for therapeutic purposes while the same antibody-linker-chelator was coupled to ^111^In for preliminary imaging and dosimetry. Ibritumomab tiuxetan was approved in 2002 for the treatment of non-Hodgkin’s lymphoma.

A similar ARC, tositumomab-^131^I, was approved the following year for the same indication. While both ARCs target CD20, tositumomab is conjugated to iodine-131, which is a β and γ emitter with a longer physical half-life of 8 days [384]. Radiolabeling of tositumomab is achieved through oxidative iodination of aromatic residues like tyrosine and histidine, rather than through chelation. The ARC was withdrawn from the market in 2014. There is debate whether this withdrawal was due primarily to a projected decline in sales related to the complexity of administration, or follow-up studies that indicated no benefit over more traditional chemo- and immunotherapies [385]. The preceding examples demonstrate the feasibility of bringing ARCs to the market, but also highlight the complexity of generating and administering radioactive therapeutics.

### 3.4. Fc Activity Engineering

Although the differentiation of the antibody is often focused on the characterization of the Fab engagement to the target epitope, not all antibodies that bind to a given target have efficient effector cell function. This was demonstrated in the comparison of different anti-CD20 antibodies that had different epitopes and subsequently different levels of effector functions [386,387,388]. Here, the avidity of Fc presentation is critical for FcγR recognition of immune effector cells. Because mAbs depend on their Fc region to elicit certain immune reactions, engineering of this domain allows for tactical modification of activity as well as enhancement of the respective physicochemical properties. Sometimes, a simple swap by moving V regions into other IgG subtypes can result in greater efficacy [389,390]. However, there can be a greater emphasis on specific Fc mutagenesis to obtain a more selective IgG effector function [88,355,391,392,393]. In addition, the coupling of the Fab and Fc regions can impact the therapeutic window for the safety and efficacy of antibodies and Fc fusion proteins [394,395,396,397]. We outline several tactics to modulate Fc activity linked to FcγR for immune effector cell function and FcRn for pharmacokinetic properties. Nonetheless, it is critical to keep in mind that the Fab domain antigen binding can affect the Fc region activity via structural allostery [398,399,400]. Hence, evaluations of specific Fc mutations should be confirmed empirically.

#### 3.4.1. Mutations that Modulate Effector Function

Protein and glycan engineering can modulate effector activity of antibodies to modulate ADCC, ADCP, opsonization, internalization, trogocytosis, and CDC activity. This engineering can also be applied to Fc fusions that comprise toxins, radioactive molecules, chemotherapeutic agents, or nucleic acids for targeted delivery [392].

Site-directed mutagenesis and X-ray crystal structures demonstrate that FcγRs make contact to IgG1 Fc at P232-S239, Y296-T299, and N325-332. Notwithstanding, the residues outside of this area may be linked to conformational changes that affect the Fc–FcγR complex formation. An abbreviated list of Fc modifications is shown in Table 2. Fc region residues can be mutated to increase the binding of antibodies to the activating FcγR and/or to enhance antibody effector functions. Mutations that decrease binding include G236A, S239D, F243L, T256A, K290A, R292P, S298A, Y300L, V305L, K326A, A330K, I332E, E333A, K334A, A339T, and P396L mutations (residue numbering according to the EU index) [345,391,392,393,401]. Experimentally, combination mutations that result in antibodies with increased ADCC or ADCP are S239D/I332E, S298A/E333A/K334A, F243L/R292P/Y300L, F243L/R292P/Y300L/P396L, F243L/R292P/Y300L/V305I/P396L, and G236A/S239D/I332E mutations on IgG1.

Fc mutations to reduce binding of the antibody to the activating FcγR and subsequently to reduce effector functions can include positions: K214T, E233P, L234V, L234A, deletion of G236, V234A, F234A, L235A, G237A, P238A, P238S, D265A, S267E, H268A, H268Q, Q268A, N297A, A327Q, P329A, D270A, Q295A, V309L, A327S, L328F, A330S, and P331S mutations on IgG1, IgG2, IgG3, or IgG4. [391,402,403,404,405]. Combinations of Fc mutations for reduced ADCC on IgG1 include: L234A/L235A; L234F/L235E/D265A; V234A/G237A; S267E/L328F; L234A/L235A/G237A/P238S/H268A/A330S/P331S; or K214T/E233P/L234V/L235A/G236-deleted/A327G/P331A/D365E/L358M. Combinations of Fc mutations for reduced ADCC on IgG2 include: H268Q/V309L/A330S/P331S or V234A/G237A/P238S/H268A/V309L/A330S/P331S. Combinations of Fc mutations for reduced ADCC on IgG4 include: F234A/L235A, S228P/F234A/L235A; S228P/F234A/L235A/G237A/P238S; or S228P/F234A/L235A/G236-deleted/G237A/P238S. Hybrid IgG2/4 Fc regions with the Fc with residues 117–260 from IgG2 and residues 261–447 from IgG4 can result in having less FcγR activity. Crystal structures and simulations of IgG1σ, IgG4σ1, and IgG4σ2 Fc variants reveal altered conformational preferences within the lower hinge and BC and FG loops relative to wild-type IgG, providing a structural rationalization for diminished Fc receptor engagement [406].

An X-ray crystal structure of the C1q-Fc region shows that complement C1q binds IgG1 at D170-K322, P329, and P331 [407,408]. To enhance CDC, Fc positions mutations can include S267E, H268F, S324T, K326A, K326W, E333A, E430S, E430F, and E430T. Combination mutations that result in antibodies with increased CDC can include K326A/E333A, K326W/E333A, H268F/S324T, S267E/H268F, S267E/S324T, E345R, and S267E/H268F/S324T [359,409,410].

The ADCC activity of antibodies can be enhanced by engineering their oligosaccharide composition. Human IgGs are N-glycosylated at Asn297 with the majority of the glycans of the well-known biantennary G0, G0F, G1, G1F, G2, or G2F forms (see Figure 5). N-linked glycosylation can be removed by using the mutation N297A on IgG1, IgG2, IgG3, or IgG4. The aglycosylated species has less FcγR activity.

Antibodies produced by non-engineered CHO cells typically have a glycan fucose content of about at least 85%. The removal of the core fucose from the biantennary complex-type oligosaccharides attached to the Fc regions enhances the ADCC of antibodies via improved FcγRIIIa binding without altering antigen binding or CDC activity. Such mAbs may be produced using different methods reported to lead to the successful expression of relatively low level fucosylated antibodies bearing the biantennary complex-type of Fc oligosaccharides, such as the control of culture osmolality [411], application of a variant CHO line Lec13 as the host cell line [412], application of a variant CHO line EB66 as the host cell line [413], application of a rat hybridoma cell line YB2/0 as the host cell line [414], introduction of small interfering RNA specifically against the α 1,6-fucosyltrasferase (*FUT8*) [415], or co-expression of β-1,4-*N*-acetylglucosaminyltransferase III and Golgi α-mannosidase II or a potent alpha-mannosidase I inhibitor, kifunensine [416,417,418]. Notwithstanding, careful monitoring of antibody glycosylation is required to control the pharmacodynamics of Abs and Fc-fusion proteins [419]. Other modifications to enhance ADCC include the introduction of bisecting N acetyl glucosamine and the removal of sialic acid residues [420].

Fc-mediated effector functions are best avoided for some applications, such as targeting cell surface antigens on immune cells or when engineering bispecific molecules to bring target diseased cells within the proximity of effector immune cells [421]. In each of these cases, it is best not to stimulate unwanted cell and tissue damage or risk undesired effector cell activation, immune cell depletion, or FcγR cross-linking that might induce cytokine release through engagement of Fc-mediated effector functions [422]. The complexity of FcγR functional properties is increased by the varying densities of activating and inhibitory receptors on the different effector cell populations [406]. Likewise, since the threshold of activation can be variable with different patients, it would be prudent for safety considerations to develop antibodies with a more silent Fc region. Thus, the development of completely silent Fc regions can be critical for biologics that do not require FcγR- or C1q-mediated effector functions [88,423,424].

#### 3.4.2. Mutations that Alter Pharmacokinetics

The PK properties of IgG antibodies are largely governed by the neonatal Fc receptor (FcRn), a heterodimer of the FcRn α chain and β2-microglobulin. Initially, FcRn was known for its role in transferring IgG from maternal milk to the neonatal circulation via FcRn-expressing intestinal epithelial cells [425,426] (and later, for transferring human IgG from the mother to fetus via FcRn-expressing placental syncytiotrophoblasts [427]). Both of these transfer mechanisms require pH-dependent binding of IgG to FcRn, with strong binding at pH < 6.5 in the acidic intestine or endosome, and significantly weaker binding in blood (pH 7.4). While it was long recognized that an Fc-binding receptor might be responsible for IgG catabolism in adults [428], the identity of this receptor as FcRn was not confirmed until three decades later [429]. Differences in IgG-FcRn affinity with pH allow IgG to be salvaged from acidic endosomes of endothelial cells and recycled back to the blood, and thus to circulate longer than other proteins of a similar size. The IgG-binding site for FcRn was localized to the CH2-CH3 elbow, which overlaps the site for *staphylococcal* protein A binding [430,431,432]. With an intact binding interface at each heavy chain, a molecule of IgG can bind simultaneously to two molecules of FcRn [433].

Because FcRn is involved in lysosomal salvage and IgG serum persistence, the IgG–FcRn interaction has been engineered to modulate the PK properties of antibodies. Enhanced serum stability may be beneficial for both patients and manufacturers, as it allows for lower-level or less frequent dosing. The combination of co-crystal structures of Fc–FcRn complexes and site-directed mutagenesis may map the Fc region regions to cover L251-S254, L309-Q311, and N434-H435. Early work demonstrated that mutagenesis of IgG to disrupt FcRn binding leads to profoundly accelerated IgG clearance [432]. The mutations I253A, H310A, and Q311A in the CH2 domain, and H433A and N434A in the CH3 domain, led to two- to five-fold decrease in the β-phase half-life of mouse IgG1 Fc fragments. Subsequent work verified the feasibility of strengthening FcRn interaction at low pH, which could extend the half-lives of Fc mutants. Ghetie et al. performed random mutagenesis of mouse IgG1 residues T252, T254, and T256 coupled with phage display to isolate variants with tighter FcRn binding at pH 6.0 [434]. Their LSF mutant had 3.5-fold higher affinity for FcRn, which was driven primarily by a slower dissociation rate. The same mutant had a half-life up to 1.6-fold longer than the wild type, which translated to a 4-fold increase in exposure. These studies suggested a potential correlation between endosomal FcRn affinity and the half-life of IgG antibodies, and initiated a search for long-lived IgG mutants that outcompete endogenous antibodies for FcRn-mediated lysosomal salvage.

One historic IgG variant with altered FcRn binding and PK is the YTE (M252Y/S254T/T256E) mutant. Initial studies using human IgG1 antibodies showed that the YTE set of mutations significantly reduced antibody serum concentrations in mice, despite a 10-fold higher affinity to both mouse and human FcRn at pH 6.0 [435]. The authors attributed this unexpected result to a concomitant increase in FcRn binding at pH 7.4 that occurred for mouse, but not human, FcRn. Later work revealed that cynomolgus FcRn, like human FcRn, binds YTE 10-fold more tightly than wild-type IgG at pH 6.0, but not significantly differently at pH 7.4 [436]. When YTE antibody was administered to monkeys, its half-life was four-fold longer than that of the control. Thus, IgG PK may be improved by using engineering strategies to increase FcRn binding at low pH while maintaining weak FcRn affinity at neutral pH. More recently, motavizumab (anti-RSV) containing the YTE substitutions became the first Fc-engineered antibody to be investigated in humans [437]. The YTE version of the antibody had a half-life of up to 100 days, or two- to four-fold longer than that of its wild-type counterpart. Although studies with intravenous administration have not indicated a higher risk of anti-drug antibodies, there is some concern that subcutaneous administration of YTE mutants could induce immunogenicity and therefore counteract any PK benefits [438].

IgG variants with altered FcRn binding not only have altered clearance; they may also have enhanced activity due to greater exposure. For instance, Zalevsky et al. developed an IgG1 LS mutant (M428L/N434S) with an 11-fold increased FcRn affinity and 3- to 5-fold increased half-life [439]. Notably, the half-life was extended for antibodies targeting a soluble antigen (vascular endothelial growth factor) and an internalizing cell-surface receptor (epidermal growth factor receptor). Thus, clearance was positively affected even in the context of an antibody with target-mediated disposition. In mouse xenograft studies, the LS antibody increased inhibition of tumor growth relative to the wild type when dosed every 10 days. Similarly, Gautam et al. showed that the LS substitutions could increase protection of rhesus macaque monkeys when incorporated into broadly neutralizing human immunodeficiency virus (HIV) antibodies [440]. Clearly, half-life extension via Fc mutation is a strategy that can be applied to a broad range of therapeutics.

Although it is tempting to oversimplify the relation between FcRn binding and PK, it must be emphasized that enhanced FcRn binding does not always translate to a longer half-life. In one informative study, Datta-Mannan et al. generated three IgG1 Fc variants with enhanced FcRn affinity at pH 6.0 and analyzed their PK profiles in cynomolgus monkeys and mice [441]. Despite up to 80-fold increases in cynomolgus FcRn affinity, the clearance of the variants in monkeys was unchanged. Furthermore, clearance was accelerated in mice even though affinity to mouse FcRn was increased almost 200-fold. As alluded to previously, the undesirable PK properties likely resulted from subtle changes in the pH dependence of FcRn binding. Borrok et al. followed up on the importance of neutral pH FcRn affinity by producing a panel of IgG1 Fc variants with variable FcRn binding at both pH 6.0 and 7.4 [442]. The authors suggest that pH 6.0 FcRn binding is directly correlated to half-life only as long as pH 7.4 binding does not also increase beyond a certain threshold. Given the sharp pH dependence required for efficient FcRn recycling, this group also proposed that half-life enhancement via Fc engineering probably cannot be improved beyond the four-fold increase already achieved.

In the Fc region, part of the CH2-CH3 domains is responsible for FcRn binding that results in recycling of antibodies for a long half-life [443,444,445,446]. mAbs with the same Fc can bind to FcRn differently, which can affect their respective PK profiles [447]. Mutations along the CH2-CH3 domains can modulate PK profiles. Single mutations that enhance the pH-sensitive binding include T250Q, M252Y, I253A, S254T, T256E, P257I, T307A, D376V, E380A, M428L, H433K, N434S, N434A, N434H, N434F, H435A, and H435R [436,439,441,442,448,449,450,451,452,453,454]. Combination mutations that can be made to increase the half-life of the antibody are M428L/N434S, M252Y/S254T/T256E, T250Q/M428L, N434A, and T307A/E380A/N434A. These mutations mediate pH-sensitive interactions with FcRn. In contrast, mutations that can reduce binding to FcRn, thereby decreasing the half-life of the antibody or Fc region molecules, can include: H435A, P257I/N434H, D376V/N434H, H435R, M252Y/S254T/T256E/H433K/N434F, and T308P/N434A. Although these mutations provide a guide, much of the PK profiles are determined empirically because of potential Fab–Fc non-covalent interactions [399].

A host of factors beyond FcRn interaction have been shown to affect the serum stability of antibodies. Especially for antibodies targeting distinct antigens or containing different variable regions, biological and physicochemical properties may supersede FcRn-binding properties in determining clearance. Even for antibodies of the same specificity, differences in variable region sequence may lead to altered biophysical properties like the charge and isoelectric point (pI), which can also affect PK. Igawa et al. observed that antibodies with lower pI values tended to be more stable in vivo. In a panel of four IgG4 molecules, the most acidic antibody (pI 7.2) had a half-life 2.4-fold longer than that of the most basic antibody (pI 9.2). As each antibody had the same constant regions and did not cross-react with mouse proteins, this result indicated that variable region sequences can cause differences in biophysical properties that affect serum persistence independent of FcRn. To further validate the observed trend, the variable regions of an IL-6R IgG1 antibody with a pI of 9.3 were engineered to generate more acidic variants (pI 6.9 and 5.5), with minor (two-fold) differences in antigen binding affinity. When administered to cynomolgus monkeys, the acidic variants had slower clearance than the wild-type antibody. Mechanistically, the decreasing positive charge may reduce attractive ionic interactions with negatively charged membranes and reduce pinocytotic cell uptake and degradation. Consistent with this explanation, engineering IgG variable regions to remove patches of positive charge (without greatly altering protein pI) has also been used to reduce non-specific binding and improve PK [455]. Likewise, the choice of framework mutations in the Fab can also influence the PK properties through differences in charge [456].

Glycosylation, glycation, and charges in the Fab region are also important for the PK properties of a mAb. FcγR expressed on the surface of blood and liver cells can facilitate the rapid removal of circulating Abs from circulation. Likewise, glycosylation patterns can impact both PK and PD significantly [259,260].

Biophysical liabilities, such as increased hydrophobicity and decreased Fc region thermal stability Tm values, can lead to lower levels of intracellular recycling that leads to subsequent intracellular degradation Antibodies binding to internalizing receptors and certain other antigens may undergo significant target-mediated clearance (reviewed in [457]). This saturable phenomenon causes nonlinear PK, where elimination is faster at lower antibody concentrations. As the antibody concentration surpasses that of the antigen, clearance becomes slower due to the increased contribution of FcRn-mediated catabolism. Thus, the distribution and elimination of antibodies can vary greatly in cases where antigen binding leads to active transport or degradation [209,277,451,458,459,460].

Extending the half-life of antigen-binding fragments and other lower molecular weight species can include strategies, such as fusion with polyethylene glycol (PEGylation), human serum albumin or an albumin-binding group, Fc region fusion, and multimerization to be above 70 kDa [461].

### 3.5. Bispecific Antibodies

When single component targeting is insufficient, an improved therapeutic response can require agents that can engage more than a single target linked to a single mechanism of action. There can be limitations with the use of mono-specific antibody formats in that some patients will not respond to such therapy after a period of time. Because there can be crosstalk between signaling pathways, there can be the development of resistance during the progression of diseased tissue. Thus, to regulate more than one disease-causing pathway, there are extensive efforts to use bispecific antibodies (BsAbs) to improve the therapeutic profile. BsAbs are engineered antibodies that have two domains that bind to two different antigens or to two epitopes on the same antigen.

There are strong therapeutic rationales for BsAbs: BsAb can target multiple causative agents for a disease with advantages over combination therapy using antibody mixtures; immune cell redirection via BsAb crosslinking of an effector biomolecule or effector cell to a specified target; and synergy through the coupling of multiple targets [462,463]. Likewise, the ability to bind to different ligands can exhibit an increased avidity and target residence time when both domains can bind simultaneously to their target sites [321,464]. This is because the binding of one binder forces the second tethered binding arm to stay close to its corresponding site. This ‘forced proximity’ favors its binding and rebinding (once dissociated) to that site. However, rebinding will also take place when the diffusion of freshly dissociated ligands is merely slowed down. Such targeting of multiple signaling pathways plays unique roles in the control of potential resistance mechanisms that are typical of the pathogenesis of various cancers. A single agent BsAb can have the advantage over a combination of mAbs by having improved compliance and less complex regulatory hurdles.

There are three approved BsAbs: Catumaxomab that can bring T cells or T lymphocytes via CD3 binding closer to cells expressing EpCAM (Trion Pharma); blinatumomab that also has a CD3-binding arm to B lymphomas with CD19(Micromet/Amgen); and Helimbra or emicizumab-kxwh (Roche—Chugai) that mimics the cofactor VIII for patients with hemophilia A. Catumaxomab is produced using quadroma technology where the HC and LC fragment of mouse mAbs against CD3 and rat mAbs against EpCAM were secreted by fusing the respective hybridomas to form a BsAb with an intact Fc. Although quadroma molecules can be produced using a variation of the hybridoma technology, Good Manufacturing Practice (GMP) scaleup to isolate the BsAb was difficult because of the challenge of isolating the BsAb from the permutations of HC/LC fragments. This BsAb was also designed to have the Fc region that can bind to FcγR-activating receptors to permit co-localization of cells with Fc receptors, such as macrophages, dendritic cells, and natural killer (NK) cells [465,466]. While the catumaxomab Fc region can enhance activation of the patient immune system against tumor cells via T cell-mediated lysis with ADCC and ADCP, there were strong adverse effects coming from the induction of anti-drug antibodies that bound to the combination or individual mouse and rat mAb sequences. Unfortunately, the formation of anti-drug Abs against the mouse and rat mAbs led to an immune response against the BsAbs, resulting in worsening of the patient’s prognosis [467].

To bypass the challenges of having mouse and rat Fc sequences, Blinatumomab was developed using the Diabody technology with a binding domain against CD19 on B cell lymphomas and CD3 binding to the surface of T cells for use in lymphoma and leukemia [468,469,470]. As with Catumaxomab, blinatumomab fosters the redirection of T cells to tumor cells without the constraints of the T cell receptor–major histocompatibility complex restrictions. The molecule is very potent and has to be delivered at low concentrations. Since the CMC process employed a recombinant bacterial expression of a single gene product, there was optimization of the downstream process to generate a stable drug substance free from residual impurities, such as host cell protein and DNA. However, because of the rapid clearance due to the short PK profile, this molecule is typically delivered via an infusion pump.

The third approved BsAb is Helimbra or Emicizumab-kxwh that have Fab regions that bind enzyme factor IXa (FIXa) and the substrate factor X(FX) [471]. Coagulation factor VIII (FVIII) can be added to reduce the bleeding complications of patients with hemophilia A. However, FVIII has poor PK properties. Thus, emicizumab was made to bind simultaneously the enzyme and substrate to mimic the partial function of factor VIII and restore some anticoagulant activity [472,473]. Humanized Abs that bind to FIXa and FX were put into a stabilized human IgG4 BsAb subtype with two sets of mutations. The BsAb has an S238P substitution to stabilize the hinge regions, preventing Fab-arm exchange. To generate the BsAb, a mixture of four expression vectors encoding the respective heavy and light chains of the FX and FIXa-specific Abs is used. The BsAb also had the “knobs-into-holes” mutation to promote the heterodimerization efficiency of the two heavy chains. Nonetheless, significant downstream purification efforts were required to isolate the BsAb. These difficulties in the manufacturing the BsAb were overcome by re-engineering the BsAb to have a common light chain for the anti-FX and anti-FIXa heavy chains and to modify the HC to facilitate ion exchange chromatography for BsAb purification [474].

We review different applications of how BsAbs can overcome the challenges of single target mAbs by modulating more than one pathway simultaneously, redirecting immune cells to specific targets; facilitating transport across tissue barriers; and delivering payloads to more specific targets.

Enhanced avidity has been reported for an EGFR x cMet BsAb (the use of EGFR x cMet refers to the BsAb, as compared to EGFR + cMet, which is the combination of the two parental EGFR and cMet mAbs) that is superior to the combination of EGFR mAbs and cMet mAbs (EGFR + cMet) [475,476]. This BsAb targets multiple resistance factors simultaneously by inhibiting primary or secondary mutations of the EGFR and cMet pathways [477]. Alternatively, a BsAb can target two non-overlapping epitopes of a target antigen to enhance the specificity and affinity of the therapeutic Ab. The bispecific binding can induce Her2 receptor crosslinking, which can further suppress Her2 activity [478].

There is great interest in utilizing Ab modulation of protein–protein interactions for diseases in the brain. However, the aforementioned pharmacokinetic properties of an IgG can prevent the high flux diffusion across tissue barriers. There are many researchers developing BsAbs to cross the blood–brain barrier (BBB) to target pathogenesis mediators in neurological diseases [479,480]. Couch et al. designed a bsAb that binds to transferrin receptor (TfR) and β-site APP-cleaving enzyme 1 (BACE1) to facilitate diffusion across the BBB [481,482]. TfR is highly expressed on the surface of the brain endothelium. After binding to TfR, the circulating bsAb is transported into the brain via receptor-mediated transcytosis. In the brain, BACE1 is an aspartyl protease that contributes to the pathogenesis of Alzheimer’s disease. Targeting BACE1 has been a strategy for treating Alzheimer’s disease [483]. The affinity between the anti-TfR Fab and TfR was selected to be weak to allow bsAb release from the endothelium and enter the brain to target the disease mediator BACE1 with the other binding arm. The safety liabilities of TfR-bispecific antibodies that cross the blood–brain barrier involves modulation of the epitopes that control the binding, internalization, and transport [481]. So far, a preclinical study showed that the BsAb could alleviate brain disease syndromes [483].

There is also interest in having molecules with asymmetric Fc regions that have different levels of engagement to FcγR and FcRn. Targeting IgG Fc region-binding selectivity of FcγRIIa versus FcγRIIb resulted in having increased ADCC activity with less other immune effector functions [484]. Asymmetric Fc with mutations in the hinge and CH2 domain can reduce binding to FcγR and C1q to decrease ADCC and CDC depletion of target cells [485]. In addition, such a strategy was used to select for Fc-silencing mutations that retain IgG1 Fc region stability to maximize CMC success. Likewise, asymmetric Fc engineering was used to have selective FcγRIIIa binding to obtain higher ADCC activity with minimal changes to IgG1 Fc stability [356].

**Figure 9:**
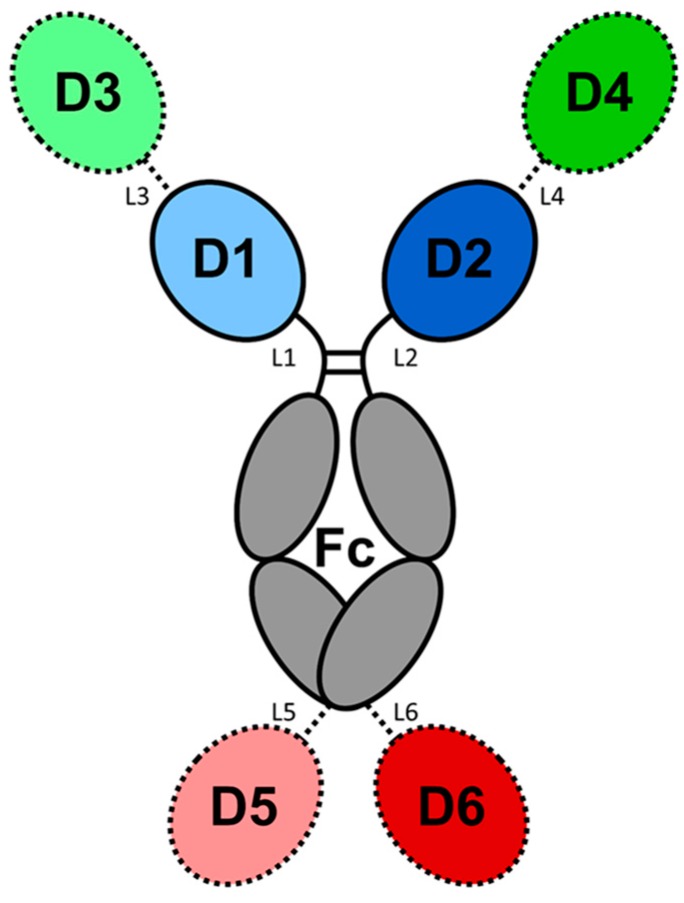
Schematic representation of bispecific and multispecific molecules. Domains 1–6 (D1–D6) can represent binding domains that can include Fab, scFv, DART^®^, VHH, and other alternative binding motifs. Linker sequences (L1–L6) can represent distinct linker regions. The Fc region can represent the IgG Fc region or be replaced with another other motif for modulation of the FcγR, FcRn, and PK profile. A standard mAb has D1 = D2, L1 = L2, and Fc = IgG Fc region.

As demonstrated in the three aforementioned BsAbs, the design was based on meeting the therapeutic hypothesis with the added challenge of meeting the CMC requirements. In the past 20 years, numerous designs can be gleaned from extensive reviews that cover many aspects of the bispecific molecule engineering, activity, and patent survey [1,486,487,488,489,490,491]. A reductionist view of bispecific agents has relied on the basic design principles of a human mAb, which include binding domains, hinge sequences, and the Fc region as shown in Figure 9. Protein engineering has extended the binding domains to include Fab, scFv, DARPins, Centyrins, Ankyrins, VHH, cytokines, enzymes, etc. The hinge has been extended to include linkers of peptide sequences from mAb sequences, subtype hinge sequences, variation of peptides having flexible linkers (Thr, Gly, Ser, Ala motifs), and rigid linkers (Pro, Arg, Phe, Thr, Glu, Gln motifs), etc. [492]. The Fc region can include the fusion of albumin-binding domains, polyethylene glycol polymers, etc. [86,397].

In this view, there are five groups of BsAb formats as shown in Figure 9: Asymmetric IgG molecules with heterodimeric heavy chains (D1 ≠ D2, with L1 = L2); fusion of IgG-binding domains (combinations of D1 with or without D3, D2 with or without D4 with L1, L2, L3, and L4); fusion of binding domains to IgG molecules (combinations of D1, D2, D3, D4, D5, and/or D6 with L1, L2, L3, L4, L5, and/or L6); engineering binding domains of IgG molecules (multiple binding at D1, D2, or Fc regions); and chemically coupled IgG fragments. A normal IgG molecule has D1 = D2 and L1 = L2 with a single Fc region (Figure 1). The continuing evolving BsAb results in changes in: Valency of binding arms to control avidity; architecture via the design of binding arms and linker types to control flexibility for access and functional activity; inclusion of different binding arms that can permit the engagement of different epitopes; and pharmacokinetic control by using Fc regions or other binding arms to serum proteins. There are many variations of tethering of binding domains and PK modulation domains that employ non-Ab motifs. However, to limit the focus on BsAb, in this review, we focus on the structure–function impacts of bispecific IgG fragments and bispecific intact IgG molecules that use Fab and Fc components.

#### 3.5.1. Bispecific Fragments

The variable region of the antibody Fab region is the smallest unit of an antibody that possesses antigen-binding capabilities. A major advantage of using fragments relative to full-length IgG is the potential for increased penetration into malignant tissue due to the decrease in size [493]. Although the absence of the Fc region abrogates FcγR binding and broadly eliminates ADCC, ADCP, and CDC, the incorporation of effector cell specificity (e.g., anti-CD3) allows for tailored effector mechanisms like T cell redirection. Similarly, FcRn-binding capability is removed. In cases where transient drug exposure is favorable (such as diagnostics), this apparent defect can lead to a desired increase in clearance. On the other hand, the half-life can be prolonged by incorporating albumin-binding functionality into one of the antigen-binding domains [494].

##### Fusion of Antigen-Binding Fragments

Perhaps the most obvious antigen-binding region of antibodies is the Fab region, which constitutes the light chain (VL and C_L_) and heavy chain Fd (VH and C_H_1). Bispecific tandem Fabs are more difficult to form than single-chain Fv and sdAb fusions, since the multiple chains may pair incorrectly to create non-functional paratopes. To address this challenge, Wu et al. made use of mutations at the LC–Fd interface that favor correct polypeptide pairing [495]. Their tandem Fabs were created using one polypeptide of linked heavy chains (VHA-C_H_1A-(G_4_S)_3_-VHB-C_H_1B) and two separate light chains (VLA-C_L_A and VLB-C_L_B). After expression in HEK cells and purification of the His-tagged protein, the EGFR × CD3 Fab was found to have similar antigen binding to the corresponding tandem scFv protein as well as better thermal stability and less aggregation. Interestingly, size had a significant impact on the ability of different bsAb formats to mediate T cell killing of EGFR-expressing cells. The 50-kDa tandem scFv had the highest potency, followed by the 100-kDa tandem Fab, and the 150-kDa bispecific IgG had the highest half-maximal effective concentration (EC50). Clearly, the size and geometry of bispecific molecules significantly impacts their ability to form a productive immunological synapse.

Fab regions can also be genetically fused to smaller binding moieties to generate bi- and trispecific molecules. For example, a human placental alkaline phosphatase (hPLAP) × BCL1 bibody (~75 kDa) was generated by fusing a BCL1 scFv to the LC C-terminus of an hPLAP Fab using a six-amino acid linker [496]. Likewise, a hPLAP × CD3 bibody was formed by fusing a CD3 scFv to the C-terminus of the hPLAP Fd using a (G_4_S)_3_ linker. More impressively, a 100-kDa tribody targeting all three antigens was generated by combining the LC (hPLAP)-scFv (BCL1) and Fd(hPLAP)-scFv (CD3) chains. While monomeric or dimeric LC contaminants were formed, the majority of antibody products had the expected composition after HEK expression. Simultaneous binding was demonstrated for each pair of tribody antigens (hPLAP × BCL1, hPLAP × CD3, BCL1 × CD3), as was T cell recognition of both tumor cell types. In addition to trispecific agents, the authors suggest that the Fab-(scFv)_2_ format could also be used to create bispecific molecules that are bivalent for one antigen.

The VHH domain of camelid heavy chain antibodies represents another compact moiety that can be tethered to Fab fragments. Li et al. prepared 65-kDa bispecific proteins called S-Fabs by fusing a VHH domain targeting CEA to the C-terminus of the Fd chain of an anti-CD3 Fab [497]. The construct was produced in *E. coli* via transformation with the normal anti-CD3 LC and the Fd (CD3)-VHH(CEA) fusion and purified using the His tag at the LC C-terminus. Interestingly, binding of S-Fab to CEA-expressing cells was achieved despite direct fusion of the anti-CEA VHH N-terminus to the anti-CD3 Fd without a spacing linker. It is not clear whether this design choice impacted the affinity of the VHH for CEA due to steric or conformational constraints. Regardless, the S-Fab depleted CEA-bearing tumor cells in a T cell-dependent manner, demonstrating the utility of these bispecific Fab-VHH fusions.

##### Fusion of Single-Chain Variable Fragments

Because the Fv (VH + VL) represents the minimal structure of human antibodies containing an intact antigen-binding interface, the scFv format has been widely used to prepare small bsAb frameworks. One of the simplest ways to generate multifunctional agents is to genetically fuse two scFv domains with differing specificity, creating tandem scFvs or scFv_2_s. Reports of such constructs date back to the 1990s. In one early study, scFvs targeting L6 tumor-associated antigen and human CD3 were genetically fused by a 27-amino acid helical linker peptide and expressed in COS cells [498]. The bispecific construct was found to co-localize T cells and L6-expressing target cells, and to elicit cytotoxic activity against the target cells. Notably, this particular scFv construct was also fused to the Fc region as a purification tag. Within a week, another report was published describing a bispecific scFv_2_ targeting fluorescein and single-stranded DNA [499]. This protein, in contrast to the previous example, was expressed in *E. coli* and refolded from inclusion bodies. In this case, the linker between individual Fvs was based on the CBH1 peptide from *Trichoderma reesi* cellobiohydrolase I. An assessment of the binding affinity demonstrated that bacterially expressed bispecific tandem scFvs can bind their antigens with a similar affinity to the parental scFv domains. These studies established that genetic fusion of distinct Fvs is a valid approach to achieve specificity for multiple antigens.

Within the realm of cancer immunotherapy, a common approach to generate a robust anti-tumor response is to co-localize effector cells to the site of malignancy and modulate their response. Bispecific T cell-engaging (BiTE) antibodies were developed shortly after the first reports of bispecific scFV_2_ and accomplished this feat by binding to both T cells (often via CD3ε) and target cell antigens with their scFv domains. In one early example, Mack et al. expressed BiTEs targeting CD3 and EpCAM in CHO cells and purified them via a C-terminal His tag [500]. Their 60-kDa protein used the VLA-VHA-VHB-VLB domain order, with a flexible G_4_S or (G_4_S)_3_ linker joining the individual Fv fragments. In addition to binding both antigens with similar properties as the parental scFvs (as demonstrated by FACS and ELISA), nanomolar concentrations of the BiTE were sufficient to elicit T cell-mediated lysis of EpCAM-expressing cells in a ^51^Cr release assay. A CD19 × CD3 BiTE with a similar construct design and expression/purification strategy was later generated and shown to have potent activity against CD19-positive lymphoma cells [469,470]. This molecule became blinatumomab, which was approved in 2014 for the treatment of acute lymphoblastic leukemia, thus demonstrating the aptitude of the tandem scFv/BiTE framework for treating cancer.

Similar to tandem scFvs is the diabody format, which contains two separate polypeptide chains (e.g., VHA-VLB and VHB-VLA) that associate non-covalently into a functional bispecific molecule. Use of a short linker between the VH and VL domains of a scFv prevents intrachain association between domains and instead favors dimerization with another molecule of scFv. By co-expressing two distinct VH-VL scFvs containing short linkers in *E. coli*, Hollinger et al. were able to form diabodies targeting both phenyloxazolone-bovine serum albumin and hen egg lysozyme [501]. Simultaneous binding of both antigens was shown by sandwich ELISA and surface plasmon resonance (SPR). In general, 15-residue linkers were found to promote the formation of scFv monomers, which can form intrachain associations, while shorter five-residue linkers tended to cause more dimerization for the formation of two antigen-binding sites. From here, strategic ordering of VH and VL domains to place half of each functional Fv in a different polypeptide chain allows for the formation of diabodies that can bind two distinct antigens. It is important to note that even when functional heterodimers are energetically preferred, non-functional homodimers may also form and must be removed via affinity chromatography.

Numerous diabody derivatives have been explored that attempt to mitigate the challenges associated with the non-covalent association of two separate chains. One straightforward way to stabilize proper chain pairing is to link the chains together, as was done for single-chain diabodies (scDbs) [502]. In contrast to scFv_2_ antibodies, which place paired domains in close proximity (e.g., VHA-VLA-VHB-VLB) and have sufficiently long linkers within an Fv, the scDb design uses a staggered domain order (e.g., VHA-VLB-VHB-VLA) and a short intra-Fv linker to prevent mismatched chain pairing. Here, 58-kDa mammalian-expressed CEA × β-galactosidase scDbs successfully recruited the enzyme to the target cells, which allowed for local activation of a prodrug to cytotoxic dauromycin. Additionally, the single-chain format had superior stability in serum than the corresponding diabody based on the retention of enzyme recruitment. Thus, consolidation of the diabody framework into a single chain can simplify protein expression and prevent chain mispairing while permitting a geometry that is distinct from that of structurally distinct scFv_2_s.

In addition to single-chain molecules, other strategies to stabilize the correctly formed diabody (especially relative to inactive homodimers) have been explored. Similar to the knob-into-hole (KiH) idea that allows for heterodimerization of half-antibodies to form intact bispecific IgG antibodies, Zhu et al. used different sets of KiH mutations to stabilize diabodies targeting HER2 and CD3 [503]. Their variant v5 (VH V37F/L45W and VL Y87A/F98M) increased bsDb purity from 72% to 92% while reducing expression yields in half. The mutations also impacted antigen binding, with HER2 affinity decreased but T cell affinity increased. The same group reported a disulfide-stabilized mutant (VH D101C and VL L46C) that increased heterodimer purity to 96%. However, this variant was difficult to produce in *E. coli*, forming insoluble aggregates and purified products that were only 65% disulfide stabilized. Shortly after, another study presented a distinct disulfide-linked diabody (VH A44C and VL G100C) that showed similar disulfide oxidation issues when expressed in *E. coli*, whereas heterodimers from *Pichia pastoris* were >90% covalently linked [504].

Dual-affinity re-targeting proteins (DARTs) are a prominent class of diabody derivatives that incorporate a C-terminal disulfide bond to stabilize the correctly formed dimer species. The first DART was described in 2010 and targeted CD16 and CD32B to recruit NK cells to act on leukemic B cells [505]. Covalent stabilization of the correct heterodimer was achieved either by appending residues LGGC at the end of each C-terminus or by adding FNRGEC to one chain and VEPKSC to the other, which mimics the sequence N-terminal to a standard IgG1κ HC-LC disulfide bond. When produced in mammalian cells, the DARTs exhibited no aggregation and were stable in serum at 37 °C for weeks and in phosphate-buffered saline (PBS) at 4 °C for months. In addition to demonstrating potent ADCC against various CD32-expressing cells with EC50 values in the pg/mL range, the DARTs were protective in a lymphoma xenograft mouse model. Rational stabilization of proper chain pairing, especially using human-derived sequences, makes the DART framework an elegant advancement in the field of bispecific fragments.

TandAbs (tandem antibodies) are a diabody-based framework that have the advantage of bivalent binding to each of two antigens. It uses the same domain orientation as scDbs but favors dimerization using a short central linker. Kipriyanov et al. first described the format in 1999, revealing that the increased valency of a CD3 × CD19 TandAb allowed for higher avidity antigen interactions as a result of slower dissociation [506]. When expressed in *E. coli*, their 57-kDa construct dimerized to form the 114-kDa tetravalent species. Similar to how diabody formation is favored using scFvs with short linkers, TandAb formation was increased using a shorter central linker (12 residues versus 27) to favor dimerization. The larger size of TandAbs was also found to increase their serum half-life relative to diabodies. In a follow-up study, TandAbs were shown to accumulate more in tumors, likely due to higher-avidity CD19 interactions [507,508]. For cases where prolonged receptor engagement is important, tetravalent TandAbs may therefore be preferred over molecules with two binding sites.

##### Fusion of Single-Domain Antibodies

While the scFv fragment (composed of linked VH and VL domains, ~25 kDa) is the minimal intact antigen-binding moiety of human antibodies, camelids produce heavy chain antibodies in which the antigen-binding moiety (VHH) is a single domain of just ~15 kDa. Thus, VHH domains of different specificities can be linked to create extremely compact bispecific molecules. For example, Conrath et al. created 37-kDa bispecific tandem VHH antibodies targeting lysozyme and β-lactamase [509]. These molecules used a 29-amino acid linker derived from the llama heavy chain antibody (IgG2a) hinge and were expressed in *E. coli*. While binding was maintained for both antigens, they noted a four-fold increase in the K_D_ value for the C-terminal VHH that might be caused by interference from the linker. This study demonstrated the potential of the tandem VHH format and paved the way for similar bispecific and multispecific molecules. Likewise, VNARs and other binding domains are amenable for similar multispecific construct design.

Another study explored whether it would be feasible to join human domain antibodies (based on VH or Vκ) to create multi-functional agents. Human dAbs against *C. albicans* secretory aspartyl proteinase 2 and mannoprotein 65 were linked by a 25-residue linker to create bispecific tandem dAbs [510]. After production in *E. coli*, mono- and bispecific dAbs were purified by protein A or protein L chromatography. Although binding parameters of the parental and bispecific dAbs were not directly compared, the bispecific molecule appeared to be more effective at clearing fungal infections. The potential advantage of using dAbs based on human VH or VL is the reduced risk of immunogenicity that may be a concern for dAbs from other animals. However, the developability properties of VHH and VNAR frameworks may be superior. Unlike the human VH/VL domains, which have hydrophobic interfaces that allow for chain pairing, VHH and VNAR domains are naturally monomeric and tend to have more hydrophilic surfaces [511].

#### 3.5.2. Fc-Dependent Bispecific Antibodies

The Fc region of BsAb Ig formats allows for applications using Fc effector function control, such as the modulation of ADCC, CDC, half-life modulation, and heterodimerization. Ideally, BsAb could be selected via taking any one set of mAb binders and mixing them with another set of mAb binders. However, the use of four expression vectors to create BsAb from two sets of HC and LC can result in a mixture of different HC and LC combinations. To minimize the downstream efforts to isolate the BsAb, there have been two general approaches—HC and LC dimerization control.

##### Heavy Chain Heterodimerization

When forming IgG-like bispecific antibodies, a major challenge is that co-transfection of heavy and light chains with different specificities leads to three possible heavy chain dimers (the heterodimer and both homodimers), where the desired heterodimer makes up only 50% of the products by random chance. As a result, about half of the protein products will be contaminating homodimers that are monospecific instead of bispecific. This has been referred to as the heavy chain problem. Several engineering strategies have been applied to drive a preference for heavy chain heterodimer formation and allow for cleaner bispecific antibody preparations.

A pioneering approach to address the heavy chain problem was to engineer ‘knob’ and ‘hole’ mutations into complementary heavy chains of distinct specificities. Ridgway et al. identified residues in the IgG CH3 domain that form direct interchain contacts [512]. Amino acids at the center of this interface were rationally mutated to introduce a protruding knob with a large surface area into one heavy chain, and a sunken hole with small sidechains into the complementary heavy chain. Using this strategy, the knob mutation T366Y and hole mutation Y407T were developed. When one mutation was incorporated into an anti-CD3 heavy chain, and the other was incorporated into a CD4-IgG immunoadhesin, the co-expression of the anti-CD3 heavy chain, anti-CD3 light chain, and CD4-IgG genes allowed for preferential (>90%) formation of the corresponding hybrid molecule with CD3- and CD4-binding functionality.

Subsequently, Atwell et al. built upon this knob-into-hole approach to generate IgG variants with an even higher preference for heterodimer formation [513]. The T366W mutation was incorporated into one CH3 domain as the knob variant, and then a library of CH3 hole mutants was generated with diversity at positions 366, 368, and 407, which are in close proximity to residue 366 on the opposite CH3 domain. Variants that increased the stability of the CH3 heterodimer were selected by seven rounds of phage display. One pair of mutants (T366W in one heavy chain and T366S/L368A/Y407V in the other heavy chain) had near complete heterodimerization and produced complexes that were more stable than those produced by singly mutated variants (T366W and Y407A) as measured by guanidine- and heat-induced unfolding. Shortly after, these knob-into-hole mutations were combined with other mutations to introduce a stabilizing interchain disulfide bond in heterodimerizing CH3 domains [514]. The resulting sets of heavy chain mutations (S354C/T366W and Y349C/T366S/L368A/Y407V) were used to produce 95%-pure bispecific antibodies targeting HER3 and CD110. Light chain mispairing was precluded using a common light chain that formed functional paratopes of differing specificity when combined with either heavy chain. Finally, the conserved function of the mutant heterodimerized Fc region was demonstrated using HER2 antibodies that had similar ADCC activity whether or not they incorporated the CH3 mutations.

In addition to shape complementarity, electrostatic interactions in the Fc region have also been engineered to favor heavy chain heterodimerization. Gunasekaran et al. identified charge-mediated interactions at the CH3–CH3 interface and strategically mutated residues to cause repulsion of identical heavy chains and attraction of opposite heavy chains [515]. While single mutations in each chain (D399K and K409D) produced some preference for heterodimerization, a pair of double mutants (E356K/D399K and K392D/K409D) allowed for superior heterodimer purity (98%). To avoid the issue of light chain mispairing, a bispecific scFv-Fc format was devised using scFvs targeting CD3 and tumor-associated receptor tyrosine kinase (TARTK). The electrostatic steering mutations allowed for the production of CD3xTARTK scFv-Fc fusions that retained binding to both antigens and induced T cell killing of TARTK-bearing cells. The use of a common light chain to avert the light chain problem allowed for the development of the bispecific antibody emicizumab (factor IXa x factor X), which was approved in 2017 for the treatment of hemophilia A [474]. There have been extensions to the knob-into-hole and the electrostatic Fc heterodimerizations to introduce Fc mutations to have distinct isoelectric points so as to enable facile purification of the heterodimeric BsAb with minimum perturbations to Fc region Tm thermal stability [516]. These modifications have been applied to CD3 redirection and dual checkpoint blockade BsAbs.

Interestingly, the structural oddities of human IgG4, including a labile hinge enabled by S228 (proline in other subclasses) and weaker inter-CH3 interactions caused by R409 (lysine in other subclasses), allow it to undergo a process called Fab-arm exchange in vivo to create bispecific IgG4 antibodies [78]. Labrijn et al. drew from this natural phenomenon to create a process called controlled Fab-arm exchange (cFAE) for the formation of bispecific IgG1 antibodies [517,518]. In contrast to the other methods described, cFAE is performed in vitro using separately purified parental antibodies, rather than relying on the co-translational formation of heavy chain heterodimer during co-expression of the respective chains. A heavy chain containing the K409R mutation was found to preferentially heterodimerize with several heavy chain mutants having variation at positions 368, 370, 399, 405, or 407 when the chains were co-incubated in the presence of a reducing agent and allowed to swap half-antibodies. Mutation of F405 to leucine (which is present in rhesus monkey IgG4) largely favored the heterodimer, and the combination of F405L and K409R in opposite chains was pursued as a general bispecific platform. The pairing consistently allowed for >95% heterodimer formation when parental antibodies were combined with a reducing agent and exchanged. The rate constants have been determined for the mechanism of IgG1 BsAb formation [519].

Subsequent buffer exchange to remove reductant allows the inter-heavy chain disulfide bonds to re-oxidize, stabilizing the bispecific product. Importantly, the interaction between heavy and light chains is not disrupted during half-antibody exchange so that light chain mismatch is avoided without the need for a common light chain. The retention of Fc-mediated functions was verified by demonstrating normal levels of CDC and ADCC from CD20 antibodies containing the mutant Fc regions, as well as normal pharmacokinetics for a bispecific CD20 × EGFR bispecific antibody generated by cFAE.

The cFAE method has been extended to include other IgG1 and IgG2 parental Abs with K409R and L368E mutations to generate stable full-length BsAbs [520]. These methods can also use modulation of protein A binding to accelerate the generation of BsAbs [521,522].

Some Fab regions were selected for dual antigen recognition [203,523,524]. However, these constructs may not be capable of binding to two different epitopes simultaneously. To overcome this challenge, single chain Fv domains have been fused onto the termini of HC/LCs to generate dual action Fab molecules. The dual variable domain, DVD, combines the variable domains of two mAbs in tandem to form dual-specific IgG molecules [525,526,527,528]. However, careful selection of the binding arms and linker domains is required to bypass developability and immunogenicity concerns.

##### Light Chain Control

Often, in CMC development, it was found that heavy chain heterodimerization was insufficient to control LC mispairing. A method to prevent this problem is to generate BsAb that have parental Abs that have different light chains families. Kappa-lambda light chain (κλ–LC) BsAbs have the same HC and two different LCs. The production of κλ-bodies involves the co-expression of one HC and two LCs (one κ, and one λ) with different binding specificities in a single cell [529]. By using serial affinity purifications, a fully human IgG format can be prepared with scalable and more facile purification. To further control LC pairing, BsAb with common LC have been developed. The BsAb can have parental Abs that have been selected to have the common LC [530]. However, custom screening is required to find the best BsAb selection in either the common LC or κλ BsAbs.

In working towards getting any pair of mAb to prepare BsAb from four expression vectors, several methods have been explored that couple LC engineering: CrossMab technology that enforces LC domain crossover in the Fab region [531]; hydrophobic and electrostatic interactions and disulfide bonds [532,533,534,535,536,537] to minimize LC swapping for proper HC/LC pairing.

#### 3.5.3. Considerations for Selection

The choice of what platforms are driven by the desired therapeutic product profile, ability to integrate engineering platform to discovery repertoire of pharmacophores, and access to licensed technology and platforms. Regardless of the platform, one major theme of lead selection of BsAb is to utilize multiple binding arms that span different epitopes. Concomitant to each epitope, it is critical to utilize a broad library of paratopes with different affinities and potencies for target engagement. Affinity selection on a target is not enough. Selection must be based on the pharmacology that may require inhibition of native ligands, co-receptor activation, or target-mediated agonism. Thus, it is not uncommon that high affinity is not linked to molecular efficacy [30,538].

After the selection of the binding arms, there is much to be done during lead selection by probing architecture, valency, order, specificity, and potency tuning to identify the best BsAb. An example of the therapeutic product profile driving molecular design involves the design of T cell or T lymphocyte redirection to attack tumor cells that are marked with a particular cell surface antigen protein. The inclusion of the valency of epitopes can mediate the avidity for low-density receptor cell targeting. These concepts are highlighted in an excellent review of different bispecific molecules that mediate T cell cytotoxicity to diseased tissues [539]. The review describes general strategies about how to optimize target antigen potency while preventing toxicity that can occur from engagement of normal tissue antigens. Likewise, some binding arms have to be modulated to have lower affinity that manifests in changes in the kinetic on and off rates to increase the potential therapeutic index of the molecule [138]. These classes of molecules can complement the utilization of engineered T cells with chimeric antigen receptors [540].

The engineering concepts for T cell redirection molecules include screening for: Different epitopes on target cell surface membrane proteins on the T cell and target cell; internalization of cell surface targets when bound to BsAb, and potency of the molecule engagement with T cell and target cell antigens. BsAb architecture can vary the orientation of the binders, linkers, valency, functional activity, and developability [539]. The role of the anti-CD3-binding domains in BsAb with a broader range of T cell agonism has been shown to have a potential wider therapeutic index [541]. In addition, the choice of the BsAb subtype can affect T cell redirection activity [389]. As the field evolves, more exploration into novel formats with different potency and kinetics of target engagement will expand possibilities for T cell therapeutics [539]. There is ongoing clinical development to understand how best to employ T cell redirection molecules versus chimeric antigen receptors [542].

Redirection of immune cells towards diseased cells are termed as effector cells that can comprise other cells beyond T cells and include NK cells, macrophages, neutrophils, monocytes, and granulocytes [396,543] to facilitate specific killing of tumor cells. Each of the effector cells express different types of activating receptors, and a specific population can be recruited by carefully selecting the targeted trigger receptor [544,545]. Redirected cytotoxic activity has been shown, with bispecific antibodies recruiting all effector cells, including macrophages [546]. A CD47xCD19 BsAb using an innate immune checkpoint control molecule, CD47, was used to block the macrophage suppressive signals in cancer cells [547]. Although the recruitment of T cells is far more widely used because of their proliferation ability and potent cytotoxicity, there are advantages in expanding the pool of immune cell clearance of malignant cells.

The choice of the binding arm paratopes should consider the developability of the individual arm, since the inclusion of problematic domains into the BsAb format can make that CMC process more difficult. The CMC criteria regarding ease of production, liabilities in post-translational modification, solubility, and clipping should be considered. However, regarding BsAbs with higher levels of dosing, several hurdles need to be addressed: Identification of a clear architectural format that is amenable to reasonable manufacturing costs. There has been some development of strategies to improve downstream purification using differential protein A binding and anti-lambda/anti-kappa chain affinity columns [548]. Presently, several clinical BsAb have involved cell line self-assembly using common HC or LC technologies, HC/LC association using point mutations in the framework, or controlled Fab-arm exchange [1,2].

Since BsAb engineering involves putting the different domains together, careful potency and efficacy selection is required to identify the best hits. In consideration of the therapeutic hypothesis, there are still other factors that can control the in vivo potency, which include the biodistribution, PK, dosing levels, and method of administration. Hence, the choice of scaffold, linkers, production host, and scale-up processes play critical roles in the preparations of the clinical development supply. Ideally, the sequence would minimize the presence of neoepitope to minimize the development of ADA. Besides formats that are completely derived from the antibody structure, fusion proteins can also achieve multifunctionality by combining the antigen specificity of one antibody domain with another targeting protein. The domains can be coupled together using leucine zippers to generate tetravalent-bispecific Abs. Likewise, developing a system to make BsAb with minimal human neo-epitopes can be important to generate molecules for both chronic and acute indications.

## 4. Evolving Applications

### 4.1. Multispecific Molecules

A natural extension of BsAbs to is have an increased number of binding arms to create multispecific Abs that can be effective in engaging more epitopes on a target. In Figure 9, the binding domains can involve combinations of binding domains using Fab, scFv, and VHH domains, or other scaffolds. With the opportunity of mixing in cytokines and enzymes, there is no shortage of possibilities for protein engineering. Ultimately, empirical screening will be required to determine what the best architecture of such multispecific agents should be.

There is a medical need to have broadly neutralizing antibodies against highly variable pathogens, such as infectious viruses. For instance, there have been several efforts to target clades of influenza A and B separately [549,550,551,552]. However, such efforts led to therapies that could only combat the respective class of influenza strains. Thus, a strategy was developed to prepare tethered multispecific antibodies with binding domains that could inhibit all known strains of influenza A and B [553]. A multispecific molecule has been engineered to incorporate diverse camelid single domain antibodies that recognize conserved epitopes on influenza virus hemagglutinin. For instance, the multidomain Ab has enhanced virus cross-reactivity and potency to inhibit influenza activity [553]. These binding domains engage previously mapped hemagglutinin epitopes that are close together. Because of the proximity of these epitopes, there can be steric hindrances that prevent the use of a tetravalent Fab-binding arm multispecific Ab format. Thus, the research effort focused on the discovery of binding arm-based VHH domains that have smaller paratope sizes. Such a molecule was able to bind to the different epitopes, target both influenza strains A and B, and had the ability to protect against all circulating strains of the virus with enhanced cross-reactivity and potency. The molecule was designed to have an active Fc region to utilize ADCC activity that could contribute to in vivo activity [554].

Antibody therapeutics targeting solid tumors are often limited by poor accumulation in and dispersal throughout the tumor tissues [555]. Since antibody fragments are small, they can be fused with various protein to create new molecules with novel functions. Antibody fragments, such as scFvs, can be fused with enzymes to localize such activity on a target cell. For instance, human RNase has been fused to an scFv targeting HER2 to endow cytotoxic activity on human carcinoma cell lines [556]. Permutations of multispecific fusions permit modulation of the enzyme activity desired [557]. Preclinical studies have shown how these molecules can reduce tumor volume.

Prodrug activation, by exogenously administered or endogenous enzymes, for cancer therapy is an approach to achieve better selectivity and less systemic toxicity than conventional chemotherapy. Typically, activating enzymes have short half-lives in the bloodstream. By engineering a cage or protease-sensitive peptide linker to block the activity of the enzyme or drug of interest, the trojan horse technology increases the drug or enzyme half-life and can prevent the drug or enzyme from cytotoxicity on healthy cells. Thus, the strategy is to use an antibody to deliver a pro-enzyme or pro-drug to destroy a target cell.

The EGFR-binding arm of cetuximab was engineered to be have its binding domain be unmasked by enzymes found in the tumor microenvironment [558]. In the absence of EGFR-binding properties in normal tissue, this molecule was inert in systemic circulation in animal models. However, when the mask was removed by appropriate proteases, the molecule restored the antigen binding and cell-based activities of cetuximab. Thus, this strategy to increase the therapeutic index with localized activation of the molecule has been expanded to other bispecific antibody applications, including immune cell redirection. Likewise, the Ab or BsAb can be used to deliver a pro-drug that can be released in the presence of enzymes in the tumor microenvironment [559] or by the higher reducing capacity in hypoxic tumor cells [560].

Alternatively, the binding arms of BsAb can be selected to have pH-sensitive binding that is responsive only in the diseased tissue of interest. For instance, a dual-function pH-responsive BsAb has been made that binds to the tumor-specific antigen on the cell surface but not on the proteolytically shed soluble domain of that tumor antigen [561]. In one example, antibody fragments can be fused with enzymes to develop antibody-directed enzyme therapies (ADEPTs) [562]. The enzyme can convert a non-toxic pro-drug into a cytotoxic drug when it is in close proximity to target cells so as to be a therapy for oncology. There is a report of an enzymatic tyrosinase nanoreactor based on metal-organic frameworks (MOFs) that activates the pro-drug paracetamol in cancer cells in a long-lasting manner. By generating reactive oxygen species (ROS) and depleting glutathione (GSH), the product of the enzymatic conversion of paracetamol is toxic to drug-resistant cancer cells. Tyrosinase-MOF nanoreactors cause significant cell death in the presence of paracetamol for up to three days after being internalized by cells, while free enzymes totally lose activity in a few hours. Thus, enzyme–MOF nanocomposites are envisioned to be novel persistent platforms for various biomedical applications [563]. Although the key limitation has been the immunogenicity of the enzyme, the inclusion of non-immunogenic enzymes in combination with prodrugs can generate potent molecules. ADEPT has the potential to be non-toxic to normal tissue and can therefore be combined with other modalities, including immunotherapy, for greater clinical benefit [564,565].

Antibody fragments can be fused with cytokines to generate immunocytokines [566,567]. Cytokines have been used for cancer patients but have substantial side effects and unfavorable pharmacokinetic profiles. The presence of the antibody domain allows for tissue-specific localization into malignant cells. These conjugated molecules can activate the immune systems of patients when in proximity to diseased tissues. This could prevent the systemic side effects associated with systemic administration of immune system-activating cytokines.

### 4.2. Intracellular Targeting

The therapeutic efficacy of anti-cancer drugs as low molecular weight chemical agents can be poor because of the inability to inhibit protein–protein interactions effectively. Because of the limitation of a small molecule drug interaction surface area, there is a great need to develop therapeutics that can control intracellular protein–protein interactions. Antibody molecules could be selected to have the specificity and potency to modulate critical cytoplasmic target molecule biology. However, antibodies cannot cross intact cellular or subcellular membranes via passive diffusion into living cells due to their size and hydrophilicity [568]. Antibody internalization into the cell can be accomplished by taking advantage of normal receptor biology: Ligand binding causes receptor activation via homo- or heterodimerization, either directly for a bivalent ligand or by causing a conformational change in the receptor for monovalent ligand and receptor-mediated endocytosis [569]. However, there is still use of the targeted receptor-mediated endocytosis machinery [570]. Manipulation of receptor-mediated endocytosis and intracellular trafficking dynamics is typically employed in the development of antibody drug conjugates. Many attempts have been made to directly deliver antibodies into intracellular compartments that include microinjection, electroporation, and protein transfection [571,572,573]. These are very selective therapies that cannot be generally scaled up with multifocal disease targeting [574]. Thus, there is a need for obtaining antibodies that can enter specific cells and tissues without the complications of the antibody–drug conjugate engineering.

Internalizing antibodies can be obtained via direct selection of internalizing phage antibodies by incubating phage libraries directly with the target cells [575,576,577,578]. However, the major challenge in this process is to identify the antigen bound by the internalizing antibody, which can be determined indirectly using the flow cytometry of target cells, and identifying the cognate antigen recognized by tumor-specific antibodies using immunoprecipitation and mass spectrometry [579]. Nonetheless, there are reports of tumor-specific internalizing antibodies from phage libraries that exert anti-tumor effects after systemic administration [580].

Much of this effort requires target-specific selection to identify such characteristics. Therefore, it is necessary to develop “promoter agents” that help improve tumor accumulation and penetration to improve the therapeutic index of antibody-based drugs [581]. There have been efforts to increase tumor access using tumor-penetrating ligands [582]. Conjugation with protein transduction domains, which are represented by cell-penetrating peptides (CPPs), such as the HIV-1 TAT peptide, has been extensively attempted in order to facilitate the intracellular delivery of antibodies or Fc-containing molecules formatted as single chain variable fragments (scFvs), antigen-binding fragments (Fabs), and full-length IgGs [583]. Optimization of CPPs continue to be applied to Fc-containing molecules [584].

Most antibodies that enter epithelial cells via receptor-mediated endocytosis are usually retained in endosomes and are then recycled out of the cells or are degraded in the lysosomes without being released into the cytosol [585]. Likewise, most of the CPP-conjugated antibodies inherited the intrinsic intracellular trafficking of the parent CPPs, which were either entrapped inside endocytic vesicles, translocated into the nucleus, or eventually degraded in lysosomes without efficient endosomal release into the cytosol. Molecular modifications were made to enhance release from the endosomes to allow for tumor tissue penetration [586].

Structural determinants of endosomal escape have been engineered into Ab variant with an ~three-fold improved endosomal escape efficiency [587,588]. The authors have been homing into a platform technology that enables an IgG to target cytosolic proteins via an endosomal escape mechanism. The elements of the engineering include having a domain to bind to the extracellular domain that permits endocytosis, a domain that improves endosomal escape efficiency, and a domain that can bind to the intracellular target [589]. Single domain antibodies have also been similarly modified for the knockdown of cytosolic and nuclear proteins [590]. The addition of endosomal escape protein domains and cell-penetrating peptides for efficient transfection broaden the application of inhibiting sdAbs.

A general strategy for generating intact, full-length IgG antibodies that penetrate into the cytosol of living cells is still of great interest [591]. A humanized light chain variable domain (VL) that could penetrate the cytosol of living cells was engineered for the association with various subtypes of human heavy chain variable domains (VHs). When light chains with humanized VL were co-expressed with three heavy chains (HCs), including two HCs of the clinically approved adalimumab (Humira) and bevacizumab (Avastin), all three purified IgG antibodies were internalized into the cytoplasm of living cells [589]. Although these methods are successful for delivering antibodies into the cytoplasm of cultured living cells, many issues, including cytotoxicity, loss of antibody stability, and difficulty of systemic administration, remain unresolved.

Intrabodies are Ab or Ab fragments that can be expressed intracellularly for binding to an intracellular protein [592,593]. These molecules can be created by the in-frame incorporation of intracellular, peptide-trafficking signals [594,595]. Additionally, they can be developed against a variety of target antigens that may be present at different subcellular locations, such as the cytosol, mitochondria, nucleus, and endoplasmic reticulum [596]. The interaction of these molecules with their target antigen results in the blocking or modification of molecular interactions, thereby leading to a change in the biological activity of the target proteins. Because the transport of Abs into a living cell from an extracellular environment is difficult, intrabodies can be expressed within the target cell via genetic engineering [568]. Because naturally occurring Abs are optimized to be secreted from the cell, intrabodies require special alterations, including: The use of single-chain antibodies (scFvs); modification of immunoglobulin VL domains for hyperstability [597]; selection of antibodies resistant to the more reducing intracellular environment [598]; expression as a fusion protein stable as intracellular proteins [599]; or the use of virus-like particles [600]. Several preclinical studies have demonstrated favorable results, including tumor growth inhibition and downregulation of viral envelope proteins, when such therapy candidates were used against inflammation [601], HIV [602], and hepatitis [603,604], respectively. The major challenge associated with these molecules is the absence of effective in vivo methods that can deliver the genetic material encoding the intrabody to live target cells [605].

## 5. Conclusions

We have provided a review of the antibody structure and function for therapeutic applications. Different examples of the engineering antibody variable domains were discussed by using rational design that is based on the experimentally derived or modeled structural information. The Fc region has been engineered to optimize effector function, clustering, and Fc receptor engagement. In general, antibody engineering of both the Fab and Fc regions is an indispensable part of the drug development process, and as such will continue to advance as more and more antibodies are generated for therapeutic use. Despite great progress in the methods of antibody engineering over the last 20 years, new approaches are in high demand. One of the remaining goals is to improve the accuracy of computational methods, which will allow for the prediction of point mutations that improve the affinity and other properties of interest. New approaches are continually being developed to create antibody-based molecules that are superior in their potency, specificity, localization, and safety. The choice of the binding domain can be tailored to engage the relevant epitopes. Likewise, engineering to change the architecture of the binding arms, Fc regions, modulatory bispecific, or multispecific domains to achieve monovalent- or avidity-driven engagement will result in more specific and potent molecules. Thus, continual process improvements to generate sufficient quantity and purity of hits will be required to facilitate comprehensive lead selection. The great diversity in antibody structure–function studies still has much room to engineer fit-for-purpose “magic bullets” with tailored PK profiles to meet different therapeutic hypotheses.

## Figures and Tables

**Table 1:** CDR definitions in Chothia numbering.

CDR	Kabat	Chothia	Martin *	PyIgClassify **	IMGT **
L1	24–34	24–34	24–34	24–34	27–32 (M − 5)
L2	50–56	50–56	50–56	49–56 (M + 1)	50–52 (M − 4)
L3	89–97	89–97	89–97	89–97	89–97
H1	31–35	26–32	26–35 (K + C)	23–35 (M + 3)	26–33 (M − 2)
H2	50–65	52–56	50–58 (K − 7)	50–58	51–57 (M − 2)
H3	95–102	95–102	95–102	93–102 (M + 2)	93–102 (M + 2)

* Martin CDRs in comparison to Kabat (K) and Chothia (C). ** PyIgClassify and IMGT CDRs in comparison to Martin (M).

**Table 2:** Common post-translational modifications to amino acids in monoclonal antibody framework molecules.

Amino Acid Changes	Chemistry	Effect on Protein	Effect on Biology
Asn-(Gly/Ser); Asp-(Gly/Ser)	Asn deamidation, Aspartic acid isomerization	Protein degradation [220,221,222]; Tertiary changes to Ab structure [223]; Isoaspartic acid [224]; Aggregation [225]	Isomerization can affect IgG avidity [226]; Deamidation affects binding [227]; Deamidation affects PK [216]
Gln	Gln deamidation	Slower deamidation than Asn, heterogeneity and stability [228]	Biological activity on Fab and Fc *
Met	Oxidation	Presence of oxidized methionine affects charged state of proteins [229,230,231]; Methionine oxidation decreases affinity to protein A and FcRn [232]	Methionine oxidation on Fc region can modulate FcγRIIa engagement [233]; FcRn and Fcγ receptors [234]; PK [235,236]
Trp	Oxidation	Changes in Trp aromaticity [237]; color changes [238]; Effects on detergent excipients for Ab formulation [239]; Higher order structure [240]	Biological activity on Fab and Fc [241,242]
Cys	Oxidation	Cysteinylation; Hinge disulfide chemistry with Cu^2+^ ion results in hydrolysis or oxidation that can lead to cleavage of the mAb [243,244,245]	Cysteinylation in CDRs leads to loss of potency [246,247]; Changing disulfide patterns in IgG subtypes [248]
His	Oxidation	Oxidized histidine react with intact histidine, lysine, and free cysteine to crosslink IgG [249]. Oxidized histidine [250,251]	Biological activity on Fab and Fc *
Asp-(Pro/Gly)	Amide bond hydrolysis	Cleavage at aspartic acid under acidic conditions [252,253]; Clipping at CH2 domain leads to aggregation [254]	Biological activity on Fab and Fc *
N terminal Glu/Gln	Pyroglutamate formation	Cyclized N terminal glutamine [255]; Challenges with molecule comparability [256]	Biological activity [256]
C terminal truncation	Carboxypeptidase substrate	Human IgG is produced with C-terminal Lysines that are cleaved off in circulation. There can be changes in charge variation	C terminal lysine loss can enhance complement activation [257]
Glycation	Reducing sugar reaction with Lysines	Charge variants [218]; Structural heterogeneity [258]	Biological activity on Fab and Fc *
Glycosylation changes	Changes in glycosylation profiles	Glycan structure [67,69,259,260,261,262,263]; High mannose and afucosylation affect stability [264]; Sialylation [265]; Fucosylation [266]	Biological activity [267]; PK and PD [268]; Clearance [269]

* Changes to critical amino acids linked to Fab-antigen or Fc-Fc receptor binding and functions.
